# *In Vitro* Modulation of Rumen Fermentation by Microbiota from the Recombination of Rumen Fluid and Solid Phases

**DOI:** 10.1128/spectrum.03387-22

**Published:** 2022-12-08

**Authors:** Wei Zhao, Mahmoud M. Abdelsattar, Xin Wang, Naifeng Zhang, Jianmin Chai

**Affiliations:** a Institute of Feed Research of Chinese Academy of Agricultural Sciences, Key Laboratory of Feed Biotechnology of the Ministry of Agriculture and Rural Affairs, Beijing, China; b Key Laboratory of Animal Genetics, Breeding and Reproduction of Shaanxi Province, College of Animal Science and Technology, Northwest A&F University, Yangling, Shaanxi, China; c Department of Animal and Poultry Production, Faculty of Agriculture, South Valley University, Qena, Egypt; d Guangdong Provincial Key Laboratory of Animal Molecular Design and Precise Breeding, College of Life Science and Engineering, Foshan University, Foshan, China; e Division of Agriculture, Department of Animal Science, University of Arkansas, Fayetteville, Arkansas, USA; Jilin University

**Keywords:** rumen microbiota transplantation, *in vitro* fermentation, fermentation parameters, bacterial community, recombination of rumen contents

## Abstract

Rumen microbiota transplantation (RMT) can improve rumen fermentation and ruminant performance. However, due to the microbial distinction in the fluid and solid phases, the current understanding of their specific roles in RMT is insufficient. Thus, this study was conducted to determine the effects of the microbiota from the recombination of the rumen fluid and solid phases on *in vitro* fermentation. The rumen fresh fluid (FF) and fresh solid (FS) phases were collected, and FS was washed for the fresh solid washing solution (FW). The fractions of FF, FS, and FW were autoclaved to obtain autoclaved fluid (AF), solid (AS), and washing solution (AW). Then, these phases were recombined to form eight treatments: FFFS, FFAS, FFFW, FFAW, AFFS, AFAS, AFFW, and AFAW. After 24 h of fermentation, the gas production in AFFS, FFFS, and FFAS was significantly higher than that of other groups. AFAS and AFAW had significantly lower alpha diversity than did other groups. The solid phase was enriched with fiber-degrading bacteria, including Treponema, *Succinivibrio*, and *Ruminococcus*. The fluid phase was dominated by *Prevotella*, *Christensenellaceae* R-7 group, and *Rikenellaceae RC9 gut group*. The washing solution had more *Ruminobacter*, *Lachnospiraceae*, and *Fibrobacter*. Moreover, the double-autoclaved phases displayed increased abundances of harmful bacteria, as AFAS and AFAW had higher Streptococcus and *Prevotellaceae YAB2003 group* abundances. A network analysis showed that the signature microbiota in AFAS and AFAW were negatively associated with the keystone microbiota in the other groups. In summary, the recombination of the solid phase and the autoclaved fluid phase had the best *in vitro* fermentation result, which provided certain references for RMT.

**IMPORTANCE** This is the first study to systematically evaluate the *in vitro* fermentation efficiency of diets by bacteria harvested and recombined from the fluid and solid phases of rumen contents, and it took into account the effect of washing the rumen solid phase. Using “reconstituted rumen content”, this study confirmed that bacteria from different fractions of the rumen digesta resulted in different fermentation production of diets and found the characteristic bacteria in each phase of rumen contents. Our data reveal that the bacteria in the solid phase have more positive effects on the *in vitro* fermentation parameters, that the combination of the autoclaved fluid phase and the fresh solid phase have the most ideal fermentation effect, and that the autoclave process significantly influenced the microbial composition and increased the abundance of harmful bacteria. This study provides a landmark reference for the future use of rumen microbiota transplantation to improve animal feed utilization and growth performance.

## INTRODUCTION

Ruminants provide meat and milk for human beings. However, in order to meet the needs of the growing population, the world’s meat and milk production must be increased by more than 60%. The shortage of land and feed resources limits the expansion of the ruminant industry. It is vital to improve the rumen fermentation and feed utilization efficiencies of ruminants. Rumen harbors a vast and diverse microbiota composed of bacteria, methanogenic archaea, protozoa, and fungal species, which are adapted to thrive in anaerobic conditions and are responsible for converting fibrous forages into volatile fatty acids (VFAs) and microbial protein in order to meet the host’s nutrient requirements (1, 2). Therefore, manipulating rumen microbiota is an effective means by which to improve both rumen fermentation and the production efficiency of ruminants.

Rumen microbiota transplantation (RMT) is an effective approach by which to manipulate microbiota for disease treatment and performance improvement. Similar to fecal microbiota transplantation in humans, RMT, using the cud from a healthy donor animal to treat a sick recipient animal that has been exposed to botanical toxins, has been practiced for a long time, since before our understanding of the rumen microorganisms (3). The RMT can alleviate rumen acidosis by improving rumen fermentation parameters, increasing the abundances of bacterial markers, accelerating rumen homeostasis recovery, and alleviating rumen epithelium damage (4). Recently, a study confirmed that the exchange of ruminal contents between high-efficiency and low-efficiency cows altered the rumen bacterial community and improved the lactation performance of cows with low milk production efficiency (5). In an era when antibiotics are banned in animal diets, RMT is an alternative approach by which to improve the feed utilization and growth of ruminants. However, to our knowledge, studies associated with RMT are still insufficient.

The rumen contents, composed of a fluid phase and a solid phase, are rich in microorganisms (2, 6, 7). In addition, the solid phase microbiota can be divided into bacteria that either are loosely adherent to the solid particles and can be detached by washing or are firmly adherent to the solid particles and cannot easily be washed away. The microbial composition in these three partitions varies with their microenvironments (6). Microbes in the rumen fluid phase account for 20% to 30% of the total rumen microbes in ruminants, mainly including amylolytic and proteolytic bacteria of Bacteroidetes, such as *Prevotella* (8, 9). The microbiota in the solid phase, occupying up to 75% of the total microbial population, includes bacteria associated with endoglucanase and xylanase activities, such as *Ruminococcus* and *Fibrobacter* (8, 10, 11). Thus, the microbiome in the solid phase that is attached to plant particles possibly contributes to fiber digestion, whereas the microbiome in the fluid phase may play a key role in the metabolism of soluble nutrients and may transmit microbes attached to the solid phase to newly ingested feed. Therefore, it is important to explore which part of the rumen digesta (the rumen content, the fluid or solid phase, or the washing fraction of the solid phase) is most suitable for RMT to improve feed utilization and ruminant performance.

We hypothesized that different parts of rumen digesta in RMT have different effects on feed digestion. In this study, the combinations of the rumen fluid and solid phases (including the before-washing and the after-washing fractions of the solid phase) as well as their autoclaved versions were used in an *in vitro* model. The rumen fermentation characteristics and the microbial community were measured after *in vitro* incubation. This work deepened our understanding of the postfermentation characteristics and functions of bacteria from different parts of the rumen and provided a certain reference for inoculation experiments in animals.

## RESULTS

### Gas production and fermentation characteristics of the *in vitro* experiment.

**FIG 1:**
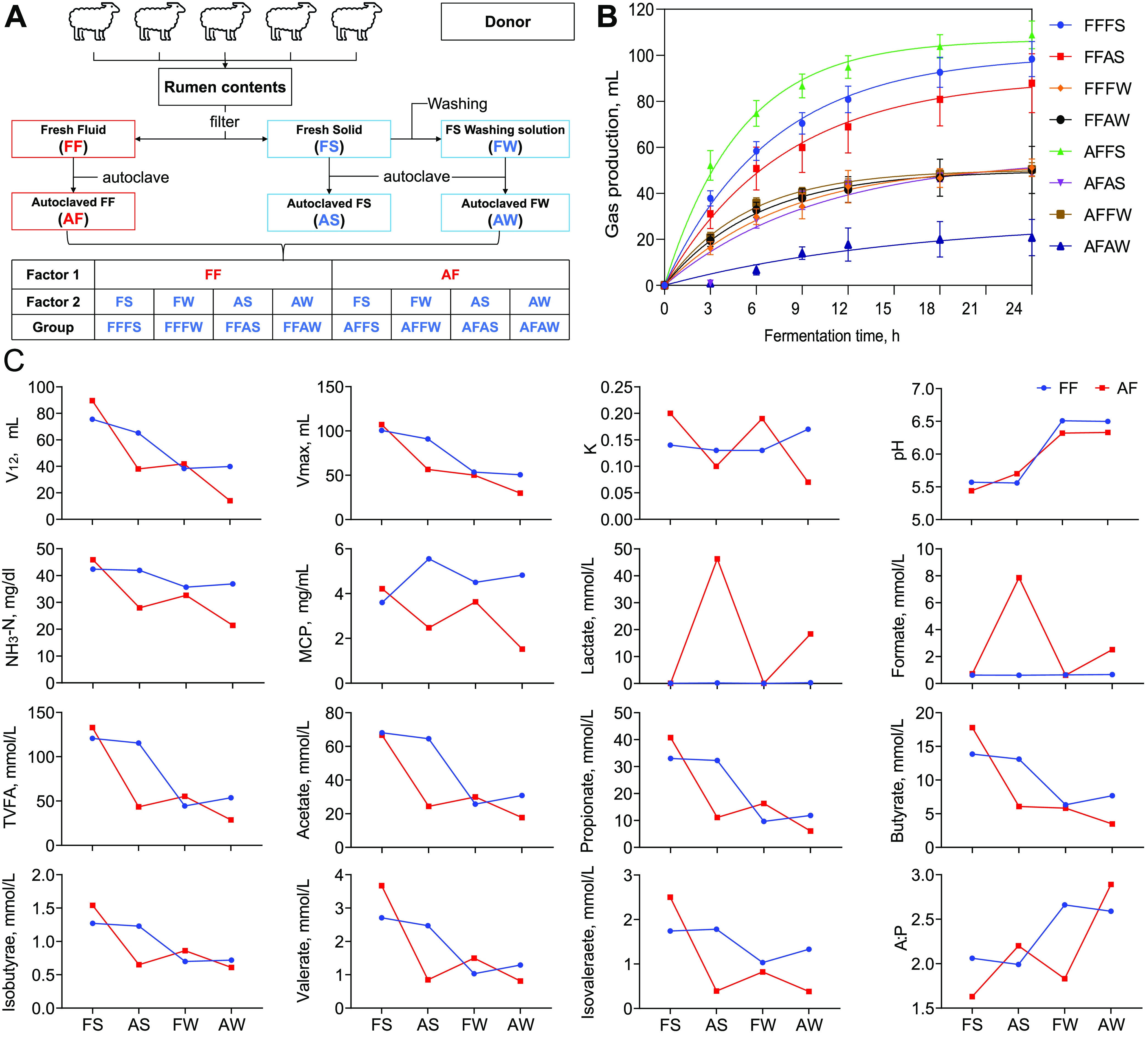
(A) The scheme of the experimental design. One factor is the rumen fluid phase, which included two levels: fresh fluid (FF) and autoclaved FF (AF). The other factor is the rumen solid phase, which included four levels: fresh solid (FS), autoclaved FS (AS), fresh solid washing solution (FW), and autoclaved FW (AW). The recombination of the levels of collected rumen fluid and solid phase were used for *in vitro* fermentation experiments. Thus, eight groups (FFFS, FFAS, FFFW, FFAW, AFFS, AFAS, AFFW, and AFAW) of different initial rumen fermentation simulation environments were obtained. (B) The effects of different combinations of rumen fluid and solid phases on gas production. (C) The effects of different combinations of rumen fluid and solid phases on fermentation parameters. V_12h_ gas production at 12 hours; Vmax, the asymptotic gas volume; K, the constant gas production rate; MCP, Microbial crude protein; TVFA, total volatile fatty acids; A : P, acetate to propionate ratio.

*In vitro* experiment, two rumen liquid phases, including fresh fluid (FF) and autoclaved FF (AF), and four solid phases, including fresh solid (FS), autoclaved FS (AS), fresh solid washing solution (FW), and autoclaved FW (AW), formed eight combinations, respectively FFFS, FFAS, FFFW, FFAW, AFFS, AFAS, AFFW, and AFAW. The temporal dynamic gas production curves of different combinations of the rumen fluid and solid phases are shown in Fig. 1B. With the extension of the fermentation time, the gas production of all of the combinations of the rumen fluid and solid phases showed an upward trend. During the whole experimental period, the gas production in AFFS, FFFS, and FFAS were significantly higher than other groups, while AFAW had the lowest gas production. There were significant differences in gas production among groups after 3 h (*P* < 0.01). The slopes of AFFS, FFAS, and FFFS were higher at 0 to 6 h. The gas production arrived at the platform after 18 h. FFFW, FFAW, AFFW, and AFAS were in the middle position and arrived at the platform 12 h later.

The effects of different combinations of rumen fluid and solid phases on gas production and fermentation parameters *in vitro* are shown in Fig. 1C and in Tables S2 and S3. The fluid phase, solid phase, and their interactions affected (*P* < 0.01) the average gas production, maximum gas production, rumen pH, NH_3_-N, lactate, formate, acetate, propionate, butyrate, isobutyrate and isovalerate, total VFA, and ratio of acetate to propionate. The solid phase and the interactions between the fluid and solid phases affected (*P* < 0.01) the average gas production, K value of gas production, and rumen valerate content. The fluid phase and the interactions between the fluid and solid phases affected (*P* < 0.01) the MCP content. Specifically, compared with the combination of FF and FS, the maximum gas production as well as the NH_3_-N, total VFA, acetate, propionate, butyrate, valerate, isobutyrate, and isovalerate concentrations were reduced (*P* < 0.01) in the combination of AF and AS as well as in the combination of FF or AF and FW or AW. The combination of AF and FS or FW increased (*P* < 0.01) the K value. The combination of FF or AF and FW or AW increased (*P* < 0.01) the pH. The combination of FF and AS increased (*P* < 0.01) the MCP content, and the combination of AF and AW decreased (*P* < 0.01) the MCP content. The combination of AF and AS or AW increased (*P* < 0.01) the lactate and formate content. The combination of AW and FF or AF increased (*P* < 0.01) the ratio of acetate to propionate.

### Bacterial diversities and composition.

A total of 2,887,921 clean bacterial 16S rRNA gene sequences were generated with an average of 78,052 reads per sample. A homogenized number of sequences of 2,592 OTUs were finally acquired.

**FIG 2:**
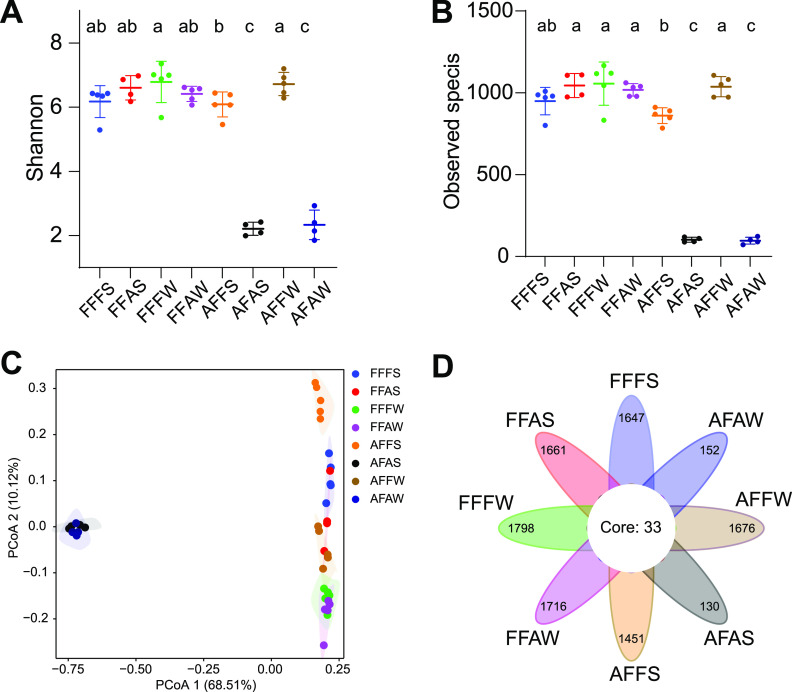
Alpha diversity of different combinations of rumen fluid and solid phases fermented for 24 h *in vitro*. (A and B) Effects of different bacteria of the rumen digesta on alpha diversity (observed species and Shannon index). (C) The beta diversity of a principal coordinate analysis (PCoA) based on Bray-Curtis dissimilarity matrices. Each point represents a unique sample. (D) Venn diagram for the composition of microbiota from fermenters among the eight groups (OTU-level analysis). FFFS = fresh fluid (FF) + fresh solid (FS); FFAS = fresh fluid (FF) + autoclaved solid (AS); FFFW = fresh fluid (FF) + fresh solid washing solution (FW); FFAW = fresh fluid (FF) + autoclaved solid washing solution (AW); AFFS = autoclaved fluid (AF) + fresh solid (FS); AFAS = autoclaved fluid (AF) + autoclaved solid (AS); AFFW = autoclaved fluid (AF) + fresh solid washing solution (FW); and AFAW = autoclaved fluid (AF) + autoclaved solid washing solution (AW).

The autoclaving process significantly decreased the microbial alpha diversity (Fig. 2A and B; Table S4). The number of observed species and the Shannon index in AFAS and AFAW were significantly lower than those in other groups (*P* < 0.05). Interestingly, although FFFS, FFAS, FFFW, FFAW, and AFFW did not show significance in either diversity or richness, they had higher observed species and Shannon index values than did AFFS (*P* < 0.05). Consistently, the AFAS and AFAW groups were obviously separated from the other six groups on the PCA plot based on the Bray-Curtis distance (Fig. 2C). AFFS showed a distinct cluster, compared to AFFW, FFFS, FFAS, FFAW, and FFFW. AFFW was also separated from FFFS, FFAS, FFAW, and FFFW. Moreover, FFFS and FFAS clustered, whereas FFFW and FFAW were closed.

To deeply integrate the similarity among the different recombinations of the rumen fluid and solid phases, a Venn plot was drawn at the OTU level (Fig. 2D). We observed 1,647 OTUs in FFFS, 1,661 OTUs in FFAS, 1,798 OTUs in FFFW, 1,716 OTUs in FFAW, 1,451 OTUs in AFFS, 130 OTUs in AFAS, 1,676 OTUs in AFFW, and 152 OTUs in AFAW. 33 OTUs (1.27% of the total) were shared among all of the groups.

**FIG 3:**
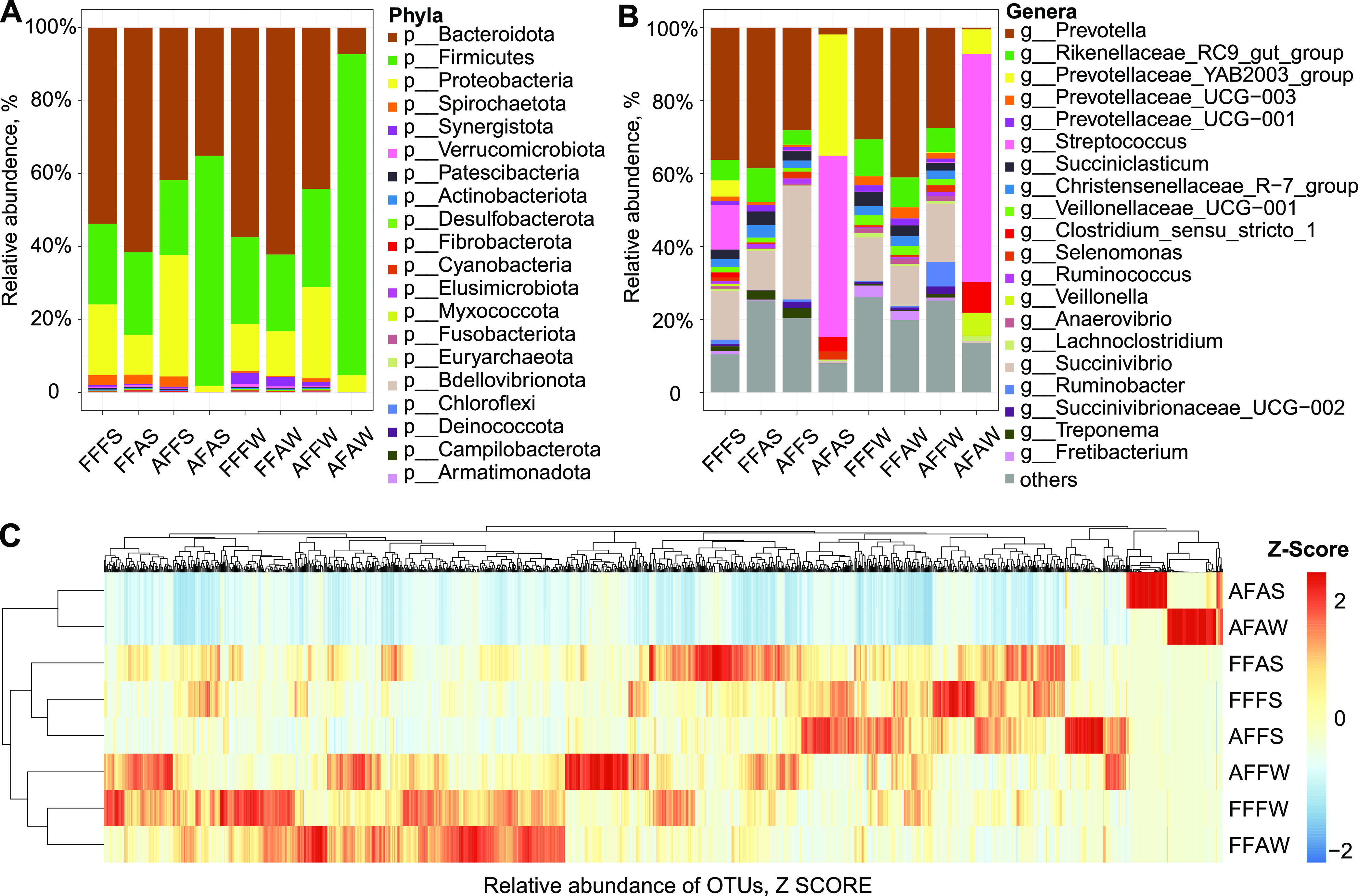
Microbial community of different combinations of rumen fluid and solid phases after 24 h of fermentation *in vitro*. The relative abundances of the rumen bacterial community at the (A) phyla level and (B) genera level after 24 h of fermentation for each group. Each column represents one group. (C) OTU heatmap, normalized by the *z* score. Each row represents a group. FFFS = fresh fluid (FF) + fresh solid (FS); FFAS = fresh fluid (FF) + autoclaved solid (AS); FFFW = fresh fluid (FF) + fresh solid washing solution (FW); FFAW = fresh fluid (FF) + autoclaved solid washing solution (AW); AFFS = autoclaved fluid (AF) + fresh solid (FS); AFAS = autoclaved fluid (AF) + autoclaved solid (AS); AFFW = autoclaved fluid (AF) + fresh solid washing solution (FW); and AFAW = autoclaved fluid (AF) + autoclaved solid washing solution (AW).

At the phylum level, a total of 20 phyla were observed, and the predominant phyla were Bacteroidetes (46.29%), Firmicutes (34.23%), and Proteobacteria (15.80%) across all samples (Fig. 3A). AF significantly decreased the relative abundances of Bacteroidetes, as its abundances were FFFS (53.8%), FFAS (61.6%), FFFW (57.5%), FFAW (62.2%), AFFS (41.7%), AFAS (35.1%), AFFW (44.2%), and AFAW (7.28%). Compared to FF, Proteobacteria increased in AFFS (33.4%) and AFFW (24.9%), whereas the Firmicutes of AFAS (63.0%) and AFAW (87.9%) increased.

At the genus level, a total of 334 genera were observed. The dominant genera, whose proportions were > 0.5%, were comprised of *Prevotella*, *Succinivibrio*, and the *Rikenellaceae RC9 gut group*, and Streptococcus and the *Prevotellaceae YAB2003 group* accounted for over 50% of the total abundance (Fig. 3B; Table S5). The autoclave fluid (AF) effect was also observed at the genus level. The abundances of *Prevotella* in AFAS (1.89%) and AFAW (0.48%) were significantly lower than those in AFFS (28.18%), AFFW (27.45%), FFFS (36.26%), FFAS (38.59%), FFFW (32.09%), and FFAW (41.08%). *Succinivibrio* showed lower abundances in AFAS and AFAW xand higher abundances in AFFS, AFFW, and FFFS. The *Rikenellaceae RC9 gut group*, *Succiniclasticum*, *Christensenellaceae R-7 group*, and *Veillonellaceae UCG-001* were similar to *Succinivibrio*. Moreover, higher abundances of Streptococcus, *Prevotellaceae YAB2003 group*, and *Clostridium sensu stricto1* were separately observed in AFAS (49.70%, 33.23%, 4.00%, respectively) and in AFAW (62.46%, 6.74%, 8.45%, respectively).

Next, a heat map with clusters based on the Bray-Curtis distances at the OTU level showed the bacterial distribution among groups (Fig. 3C). AFAS and AFAW were clustered together. FFAS, FFFS, and AFFS were under a cluster. AFFW, FFFW, and FFAW had similar compositions of OTUs. Moreover, although the groups clustered, the compositions of OTUs between groups were also different, such as those of AFAS and AFAW (Table S6).

### Signature taxa differentiating the recombinations of the rumen fluid and solid phases.

**FIG 4:**
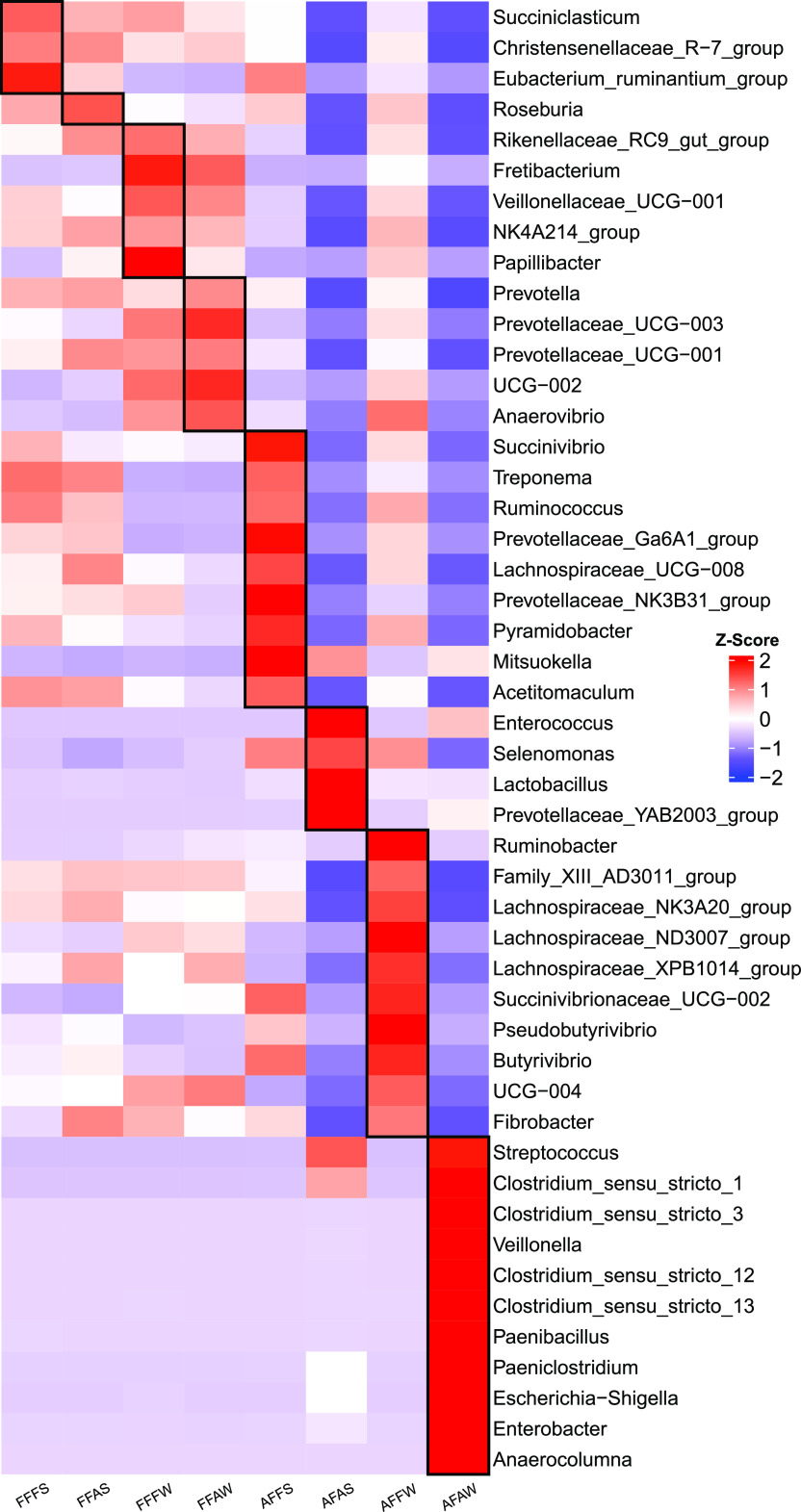
LEfSe to identify signature microbiota differentiating the recombination of the rumen fluid and solid phases after 24 h of fermentation *in vitro*. Heat map depicting the group-associated genera identified by the LEfSe algorithm, including the featured taxa of different groups with a LDA score of >3. FFFS = fresh fluid (FF) + fresh solid (FS); FFAS = fresh fluid (FF) + autoclaved solid (AS); FFFW = fresh fluid (FF) + fresh solid washing solution (FW); FFAW = fresh fluid (FF) + autoclaved solid washing solution (AW); AFFS = autoclaved fluid (AF) + fresh solid (FS); AFAS = autoclaved fluid (AF) + autoclaved solid (AS); AFFW = autoclaved fluid (AF) + fresh solid washing solution (FW); and AFAW = autoclaved fluid (AF) + autoclaved solid washing solution (AW).

A supervised classification of the microbiota among groups was carried out by utilizing the linear discriminant analysis (LDA) effect size (LEfSe) analysis, which is often used to identify the presence and effect sizes of biomarker bacteria among different groups (Fig. 4). The genera *Succiniclasticum* and *Christensenellaceae R-7 group* were enriched in the FFFS group. *Roseburia* was overrepresented in FFAS. The *Rikenellaceae RC9 gut group* was the signature for FFFW. FFAW had higher abundances of *Prevotella*, *Prevotellaceae UCG-003*, and *Prevotellaceae UCG-001*. For these four groups under FF, signatures were found for each group, but the abundances of these signatures were also higher in other FF, compared to AF. More variations of abundances of signatures existed in AFFS, AFAS, AFFW, and AFAW under AF. *Succinivibrio*, Treponema, *Ruminococcus*, and *Mitsuokella* were identified as the bacterial signature for the AFFS group, whereas *Prevotellaceae YAB2003 group*, *Selenomonas*, *Lactobacillus*, and *Enterococcus* were higher in the AFAS group. AFFW had greater abundances of *Ruminobacter*, *Succinivibrionaceae UCG-002*, *Pseudobutyrivibrio*, and *Butyrivibrio*. Streptococcus, *Clostridium sensu stricto1*, *Clostridium sensu stricto3*, *Veillonella*, *Clostridium sensu stricto13*, *Paenibacillus*, *Paeniclostridium*, and *Escherichia-Shigella* were higher in AFAW.

### Microbial interactions.

**FIG 5:**
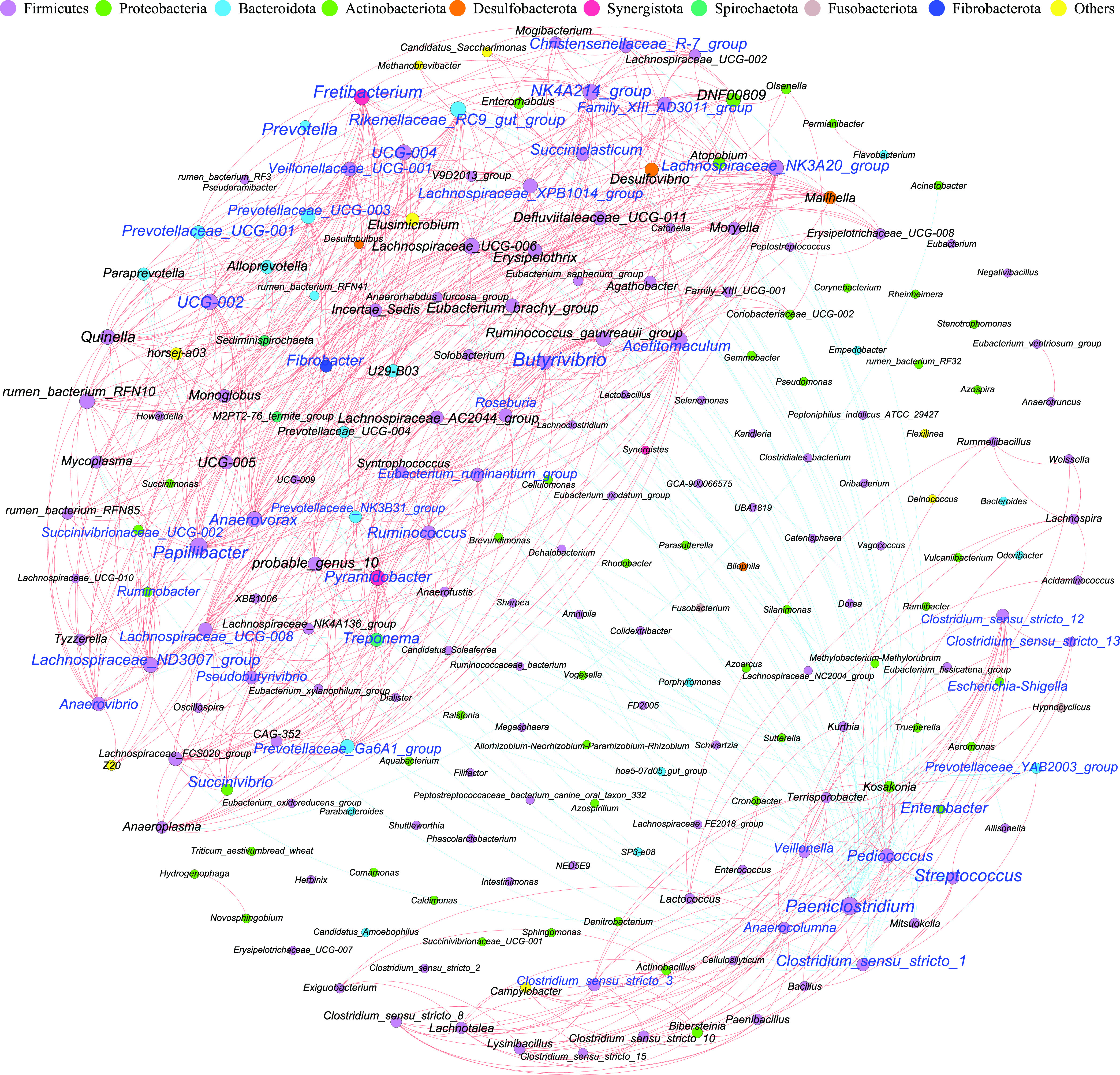
Network analysis to classify bacterial interactions. The network analysis showed the degree of correlation between the bacteria at the genus-wide level (Spearman’s |*r*| > 0.7 and *P* < 0.05). The nodes represent the phylum classification of the genus, and the node sizes represent the weights of each taxon. Nodes representing the same phylum are shown in the same color. Lines between two genus-level bacteria represent the correlation, with a red line indicating a positive correlation, a blue line indicating a negative correlation, and the line size indicating the magnitude of the correlation. Blue represents the featured taxa of different groups with a LDA score of >3.

Two subunits with negative correlations were significantly observed (Fig. 5). One of the subunits mainly consisted of the microbial signature for FFFS, FFAS, FFFW, FFAW, AFFS, and AFFW, including *Succiniclasticum* and the *Christensenellaceae R-7 group* for FFFS, the *Rikenellaceae RC9 gut group* for FFFW, *Prevotella*, *Prevotellaceae UCG-003*, and *Prevotellaceae UCG-001* for FFAW, *Succinivibrio*, Treponema, and *Ruminococcus* for AFFS, and *Ruminobacter*, *Succinivibrionaceae UCG-002*, *Pseudobutyrivibrio*, and *Butyrivibrio* for AFFW. Moreover, the signatures for AFFS and AFFW in this subunit formed distinct nodes, compared to the microbial signatures for FFFS, FFAS, FFFW, and FFAW. The other subunit was composed of the signature microbiota for AFAS and AFAW, such as *Paeniclostridium*, Streptococcus, *Pediococcus*, *Prevotellaceae YAB2003 group*, and *Clostridium sensu stricto 1*.

### Major factors influencing the signature microbiota.

**FIG 6:**
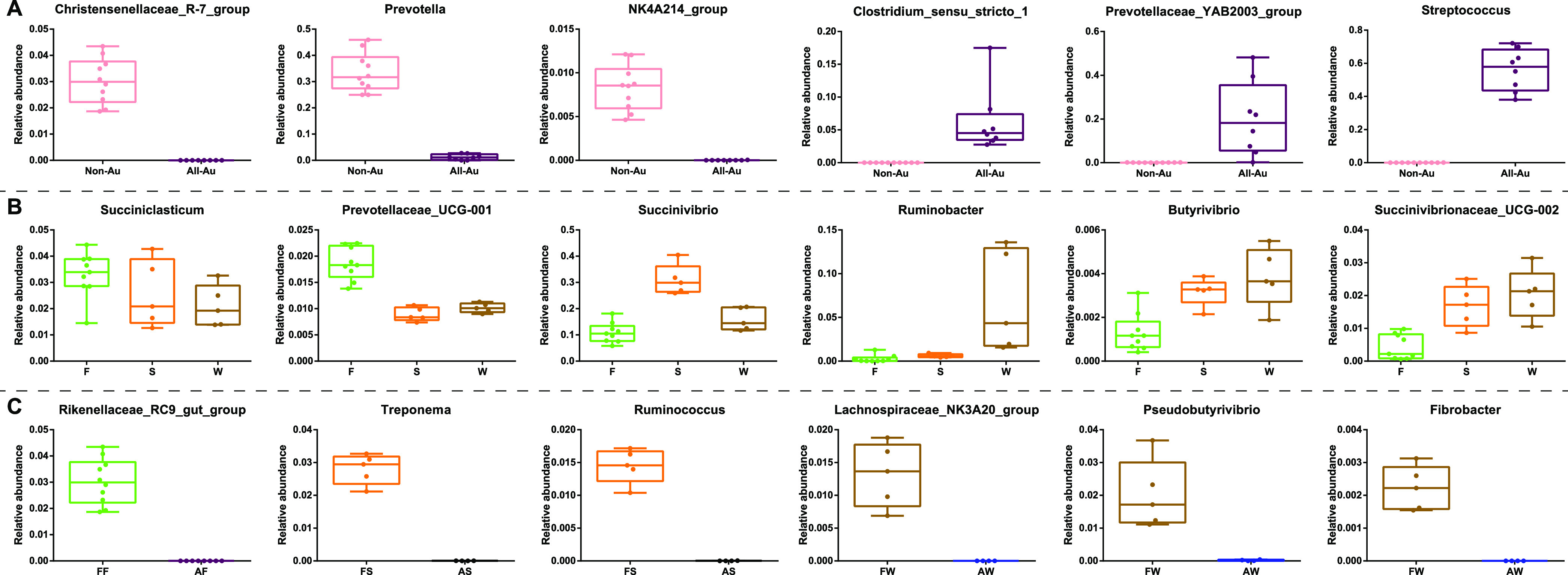
Main factors affecting the signature microbiota. (A) Nonautoclaved versus all-autoclaved. (B) Fluid phase (F) versus solid phase (S) versus washing solution (W). (C) Autoclave effect for one fraction (fluid, solid, and washing): (i) fresh fluid (FF) versus autoclaved fluid (AF); (ii) fresh solid (FS) versus autoclaved solid (AS); and (iii) fresh washing solution (FW) versus autoclaved washing solution (AW).

Based on the above analyses, major factors, including autoclaved versus nonautoclaved, solid versus fluid phase, etc., significantly impacted the microbial communities. The signature microbiota affected by the corresponding factors are shown in Fig. 6. First, regarding the autoclaved effects on both fractions, the *Christensenellaceae R-7 group*, *Prevotella*, and *NK4A214 group* were higher in the nonautoclaved group (Non-Au: FFFS and FFFW) (Fig. 6A). In contrast, *Clostridium sensu stricto 1*, *Prevotellaceae YAB2003 group*, and Streptococcus increased in the autoclaved groups (All-Au: AFAS and AFAW). Moreover, compared to the autoclaved groups, these three microbiota showed similar patterns in groups with one autoclaved fraction, such as FFAS, FFAW, AFFS, and AFFW (Fig. S1A).

Next, the comparisons between the fluid phase, solid phase, and washing solution were determined, considering that the autoclave process killed all of the bacteria in one fraction. Specifically, FFAS and FFAW were merged into absolute fluid phase groups (F), AFFS was considered solid phase (S), and AFFW was defined as washing solution (W). *Succiniclasticum* were not affected by the fractions (Fig. 6B). *Prevotellaceae UCG-001* and *Prevotella* were higher in F (Fig. 6B; Fig. S1B). *Succinivibrio* and *Mitsuokella* were specifically enriched in S, whereas *Ruminobacter* was greater in W. In addition, *Butyrivibrio*, *Succinivibrionaceae UCG-002*, and *Selenomonas* were higher in both S and W.

Then, the autoclave effects for one fraction (fluid, solid, and washing) were specifically estimated by removing the impacts of fresh fractions. Regarding the autoclave effects on the fluid phase, FFAS and FFAW were defined as fresh fluid (FF), as compared to autoclaved fluid (AF), which included the AFAS and AFAW groups. The *Rikenellaceae RC9 gut group* was higher in FF and was almost absent in AF (Fig. 6C). To determine the autoclave effects on the solid phase, AFFS was selected as the fresh solid group (FS), whereas AFAS was defined as the autoclave solid (AS). Treponema and *Ruminococcus* were higher in FS and were hardly observed in AF, whereas the *Prevotellaceae YAB2003 group* was enriched in AS (Fig. S1C). Similarly, the autoclave effects on the washing solution were also estimated. AFFW was considered fresh washing (FW), whereas AFAW was considered autoclave washing (AW). The *Lachnospiraceae NK3A20 group*, *Pseudobutyrivibrio*, and *Fibrobacter* were higher in FW but were scarce in AW. In contrast, *Veillonella* and *Clostridium sensu stricto 13* were enriched in AW (Fig. S1C).

### Relationship between bacteria and phenotypes.

**FIG 7:**
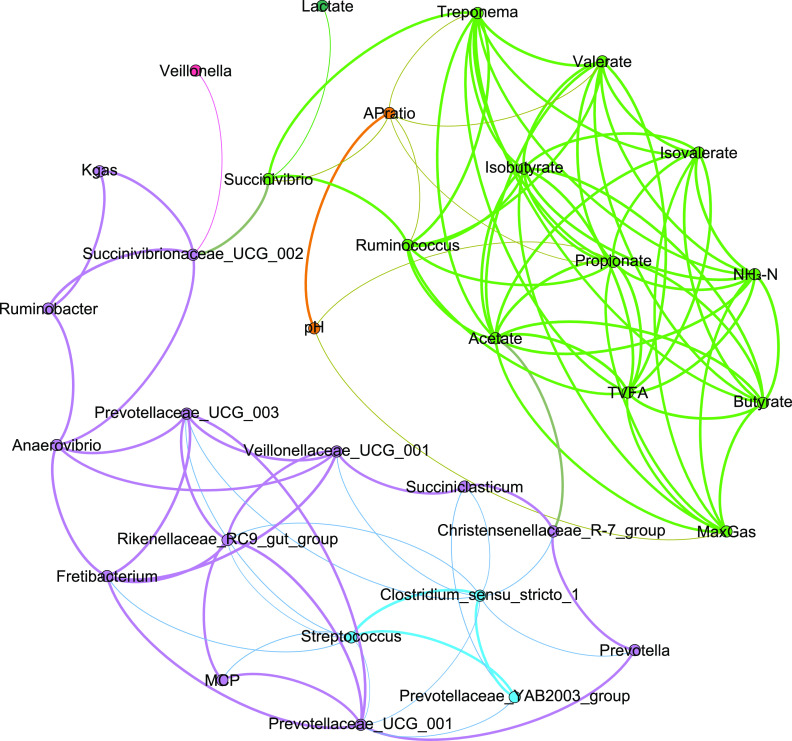
Network of rumen fermentation parameters and the top 20 genus-level bacteria. The factors that were observed as regimen-associated features and were identified as function drivers form the main subnetwork (Spearman’s |*r*| > 0.6 and *P* < 0.05). We detected six main subnetworks (shown as pink, purple, blue, light green, orange, and dark green clusters). The line size indicates the magnitude of the correlation, and the connecting line between the modules is a transition color between the two colors. TVFA, total VFA; MaxGas, asymptotic gas volume; Kgas, constant gas production rate; APratio, acetate to propionate ratio.

A co-occurrence network analysis based on the calculation of Spearman’s rank correlations revealed co-occurrence relationships between the top 20 genus-level bacteria and the fermentation parameters (Fig. 7). The factors that were observed as regimen-associated features and were identified as function drivers formed the main subnetwork. We detected six main subnetworks (shown in purple, green, blue, orange, pink, and dark green clusters). The thickness of the line represents the correlation coefficient, and the connecting line between the modules is a transition color between the two colors. In brief, the green cluster is shaped mostly by the fermentation parameters (i.e., acetate, propionate, butyrate), while another big cluster (purple) was comprised of bacteria. *Succinivibrio* and *Succinivibrionaceae UCG-002* were hub nodes connecting the green and purple clusters. In the green module, aside from *Succinivibrio*, other two bacteria, namely, Treponema and *Ruminococcus*, had a complex correlation with the fermentation parameters. *Christensenellaceae R-7 group*, as the signature for AFFS, was another hub node in purple cluster that connected to the green module. Interestingly, the *Prevotellaceae YAB2003 group* identified as the signature for AFAS, and Streptococcus and *Clostridium sensu stricto1*, which were abundant in AFAW, shaped the blue module and had a connection with the purple cluster. *Succiniclasticum*, with its high abundances in FFFS, FFAS, FFFW, and FFAW, was associated with the pH of fermented production. Additionally, the microbiota higher in FS were positively correlated with the fermentation parameters (Fig. S2).

### Functions of microbiota.

**FIG 8:**
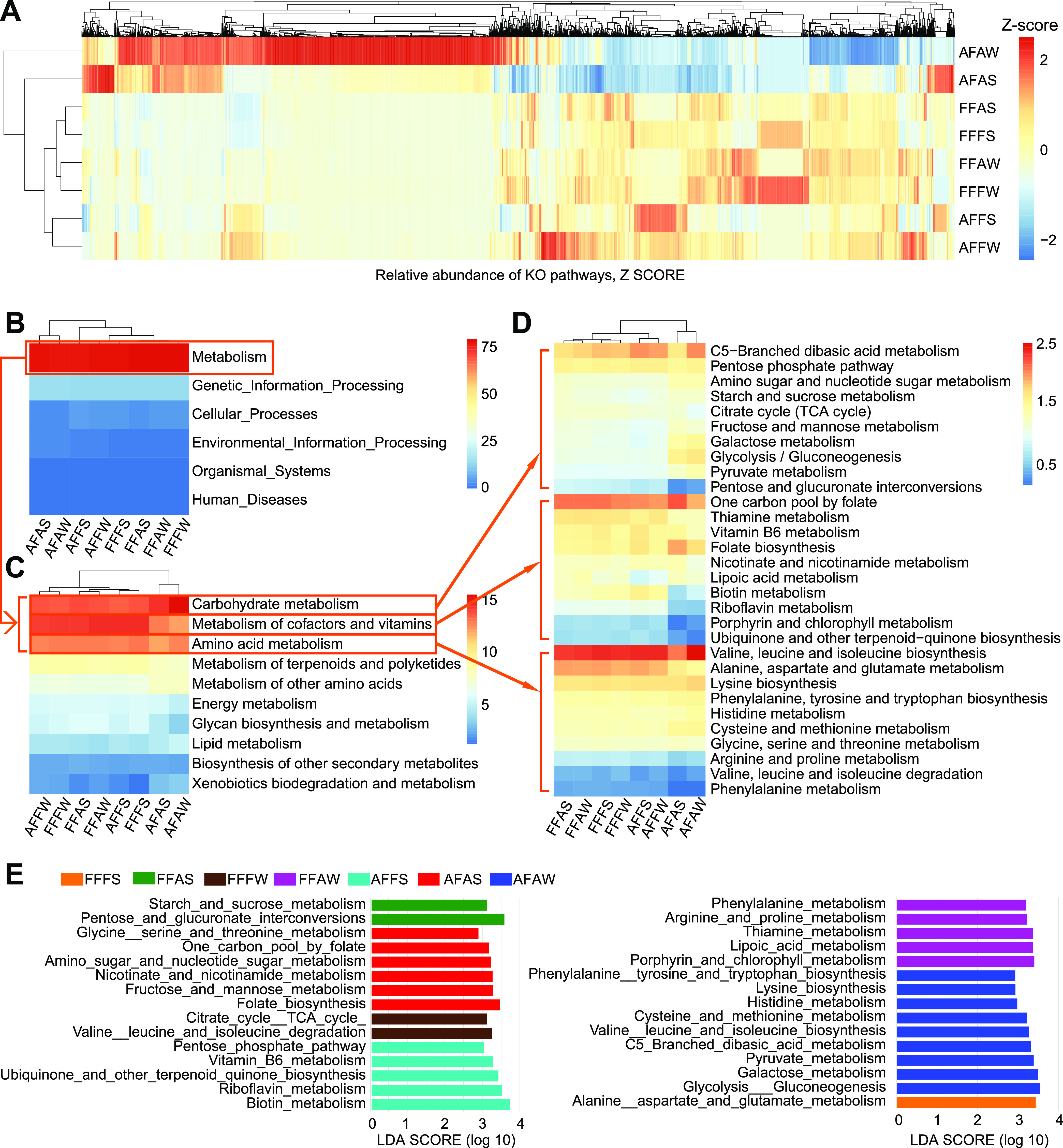
Functional predictions for microbiota from different bacteria in the rumen digesta of the *in vitro* fermentation of the diet. (A) Heatmap of KOs, *z* score. Relative abundance of function pathways: level 1 (B), level 2 (C, top 10), and level 3 (D, top 10 of Carbohydrate Metabolism, Metabolism of Cofactors and Vitamins, and Amino Acid). (E) Featured pathways of different groups by LEfSe. FFFS = fresh fluid (FF) + fresh solid (FS); FFAS = fresh fluid (FF) + autoclaved solid (AS); FFFW = fresh fluid (FF) + fresh solid washing solution (FW); FFAW = fresh fluid (FF) + autoclaved solid washing solution (AW); AFFS = autoclaved fluid (AF) + fresh solid (FS); AFAS = autoclaved fluid (AF) + autoclaved solid (AS); AFFW = autoclaved fluid (AF) + fresh solid washing solution (FW); and AFAW = autoclaved fluid (AF) + autoclaved solid washing solution (AW).

Consistent with the distribution of the OTUs, functional predictions of all groups via PICRUSt2 yielded similar clusters (Fig. 8A). At level 1, six categories, including Metabolism (78.68%), Genetic Information Processing (14.29%), Environmental Information Processing (2.06%), Cellular Processes (4.13%), Organismal Systems (0.46%), and Human Diseases (0.37%), were observed across all samples (Fig. 8B). In the Metabolism pathway, Carbohydrate Metabolism (14.00%), Metabolism of Cofactors and Vitamins (13.95%), and Amino Acid (12.73%), were the most active (Fig. 8C), and the three pathways further showed the level 3 KEGG Orthology corresponding to their top 10 abundances (Fig. 8D). A Kruskal-Wallis test confirmed that a total of 34 pathways marked in the three figures showed statistically significant differences between the eight groups (*P* < 0.05) (Table S7).

A LEfSe analysis was performed to classify the pathway biomarkers from a total of 155 KEGG level 3 pathways (Fig. 8E). The LEfSe analysis showed that the pathways related to alanine, aspartate, and glutamate metabolism were upregulated in the FFFS group. The citrate cycle (TCA cycle) as well as valine, leucine, and isoleucine degradation were enhanced in the FFFW group. Thiamine metabolism, porphyrin and chlorophyll metabolism, lipoic acid metabolism, phenylalanine metabolism, and arginine and proline metabolism were more active in the FFAW group. Starch and sucrose metabolism as well as pentose and glucuronate interconversions were significantly expressed in the FFAS group. Vitamin B6 metabolism, the biosynthesis of ubiquinone and other terpenoid-quinones, riboflavin metabolism, biotin metabolism, and the pentose phosphate pathway were abundant in the AFFS group. One carbon pool by folate, nicotinate and nicotinamide metabolism, folate biosynthesis, fructose and mannose metabolism, amino sugar and nucleotide sugar metabolism, and glycine, serine, and threonine metabolism were enriched in AFAS. Pyruvate metabolism, glycolysis/gluconeogenesis, galactose metabolism, C5-branched dibasic acid metabolism, valine, leucine, and isoleucine biosynthesis, phenylalanine, tyrosine, and tryptophan biosynthesis, lysine biosynthesis, histidine metabolism, and cysteine and methionine metabolism were more responsive in the AFAW group. The AFFW group did not display any significantly enriched functional pathways.

## DISCUSSION

This is the first study to systematically evaluate the fermentation efficiency of diets by bacteria harvested and recombined from the fluid and solid phases of rumen contents. This study highlights how fermentation parameters, bacterial composition, and function are changed by 24 h of *in vitro* fermentation. The results support the hypothesis that bacteria from different fractions of the rumen digesta result in different fermentation production of diets. Our data reveal that the bacteria in the solid phase have more positive effects on the *in vitro* fermentation parameters, that the combination of the autoclaved fluid phase and fresh solid phase have the most ideal fermentation effect, and that the autoclave process significantly influences microbial composition and increases the abundance of harmful bacteria.

Ruminal homeostasis and fermentation are the basis for the metabolism, development, and health of ruminants (12, 13). *In vitro* rumen fermentation experimentation is a technology by which to stimulate rumen fermentation and to reduce the cost of animals and related animal welfare issues by testing multiple samples in batch. Furthermore, it avoids the effects of host interference factors, such as the rumen passage rate and absorption, on the fermentation process. Therefore, this study is the starting point for the transplantation of rumen microbiota. In this study, the groups containing fresh solid phase bacteria (i.e., AFFS and FFFS) had lower values of acetate to propionate ratios and higher asymptotic gas volumes and yields of total VFA, propionate, and butyrate. The higher asymptotic gas volumes of AFFS and FFFS might be caused by increasing the substrate and bacteria in the incubation. The VFA can provide metabolic energy to supply more than 70% that needed for the growth and development of ruminants (14). The lower acetate to propionate ratios reflect the better rumen fermentation modes on the development of rumen tissue and energy utilization, which can increase the content of propionate neo-glucose and the growth performance (15). Therefore, AFFS and FFFS may achieve better fermentation production in the actual inoculated animal experiments and thereby better affect digestion, capacity utilization, rumen development, and animal growth.

Autoclaved process of both the fluid and solid phase significantly affected the bacterial community after 24 h of fermentation. After autoclaving, the original rumen bacterial community was destroyed, and the biomass and diversity were greatly reduced. Moreover, most of the bacteria that had positive effects on the fermentation parameters died out after 24 h of fermentation. However, the harmful bacteria, including Streptococcus, *Prevotellaceae YAB2003 group*, and *Clostridium sensu stricto 1*, were significantly increased. As we observed, AFAW and AFAS still produced some gas during the first 24 h of fermentation, which might be due to these harmful bacteria. The *Prevotellaceae YAB2003 group* were reported to have the ability to degrade fiber sources, such as hemicellulose or xylan (16, 17), which may be one of the important reasons for gas production. *Veillonella* enriched in the AFAW impacted beneficial, gas-producing fermentation processes by affecting the colonization of *Succinivibrionaceae UCG-002* (18, 19). Bacteria attached to diets may make important contributions to the postfermentation microbiota structure, as dominant bacteria in the rumen content, such as the *Christensenellaceae R-7 group*, *Prevotella* and *NK4A214 group*, were susceptible to autoclaving factors. At the same time, we observed that the nonautoclaved groups had higher abundances of beneficial fermentative bacteria, which means that there was a phenomenon of bacterial clustering and that two or more clusters of bacteria had a mutual inhibitory effect. This indicated significant competition between the beneficial and harmful microflora associated with the rumen fermentation products. After beneficial microbiota had taken the lead in occupying a favorable ecological niche, the reproduction of harmful bacteria could be greatly reduced, which may be due to the increased direct competition between the starch-degrading bacteria (such as *Prevotella*, *Succinimonas*, *Selenomoas*, etc.) and the fiber-degrading bacteria (such as the *Clostridium sensu stricto 1* and the *Prevotellaceae YAB2003 group* in the double-autoclaved groups) for fermentable carbohydrates, their main energy source (20).

Shared bacteria were observed among the fluid phase, solid phase, and wash solution after *in vitro* fermentation. *Succiniclasticum*, which had high and similar abundances among them, may be an important rumen microbial cornerstone for fermentation. *Succiniclasticum* increased in early fattened lamb was associated with higher ADF intake, rumen metabolism, and the production and conversion of succinic acid to propionic acid (21, 22). However, different bacterial compositions were generally found in the fluid phase, solid phase, and wash solution groups. *Prevotella*, *Rikenellaceae RC9 gut group*, *Prevotellaceae UCG-003*, and *Prevotellaceae UCG-001* were greater in the rumen fluid phase, which is consistent the results of previous studies (8, 9). *Prevotella* is a Gram-negative bacterium belonging to the phylum Bacteroidetes and is dominant across diet regimes and sampling communities (23). *Prevotella* can degrade and utilize structural polysaccharides in plants, such as pectin, starch, and xylan, but needs to be cocultured with cellulolytic bacteria (24). The *Rikenellaceae RC9* gut group may play an important role in lipid metabolism and in the process of ruminal hemicellulose degradation (25, 26). *Prevotellaceae UCG-003* can utilize branched-chain VFAs and is involved in glucose metabolism (27). Therefore, it is not surprising that the groups containing fresh fluid had better fermentation results, since the rumen fluid phase is critical for nutrient digestion. Moreover, the signature microbiota that were attached tightly to the solid phase were *Succinivibrio*, *Selenomonas*, and *Mitsuokella*, all of which had strong feed adhesion. These three are acid-utilizing bacteria that could adapt to the faster fermentation and could reduce the pressure on the body to metabolize lactic acid. Additionally, microbiota loosely attached to the solid phase were the signatures identified for the washing solution, including *Ruminobacter* and *Pseudobutyrivibrio*. They had strong feed tropism and played important roles in feed digestion, which is consistent with previous reports that *Ruminobacter* is positively associated with *Selenomonas* in degrading starch and hemicellulose (28, 29). These all imply that each fraction of the rumen content has its own primary functions in nutrient utilization and that their recombination may produce better fermentation results. Rumen microbiota transplantation should use different fractions or their combinations for different goals.

Through the analysis of the autoclave fraction, the roles of the microbiota in a specific fraction could be well-understood. We found that Treponema and *Ruminococcus* were specifically higher in AFFS, although they also presented in FFFS and FFAS. They were associated with *Prevotellaceae UCG-003* and *Prevotellaceae UCG-001* as the dominant bacteria in the rumen fluid phase. These two bacteria were associated with fiber degradation and may have antagonism to the bacteria in the rumen fluid (30–32). After the removal of live bacteria from the rumen fluid, these two bacteria may effectively use substrates from the fluid phase to ferment diets, which is an echo that AFFS had the best gas production of *in vitro* fermentation. Additionally, flagellar assembly and bacterial chemotaxis were more active in the AFFS group, meaning that the bacteria were increasing their vitality to better utilize the substrate. Rumen microbes require biotin for efficient fermentation, as a lack of biotin significantly reduces cellulose digestion and the production of volatile fatty acids (especially the propionate pathway), which are required for carbohydrate, fatty acid, amino acid, and nucleic acid metabolism, and biotin is an essential cofactor for various enzymes (33, 34). Meanwhile, vitamin B6 metabolism, ubiquinone and other terpenoid-quinone biosynthesis, riboflavin metabolism, and the pentose phosphate pathways were abundant in the AFFS group, meaning that hydrogen atom transfer and fatty acid synthesis were more active. However, the predicted functions associated with energy metabolism (galactose, starch, and sucrose metabolism) were found in FFFS. Therefore, the combination (AFFS) could be potentially used for rumen microbiota transplantation to improve feed utilization.

The co-occurrence network analysis and the network analysis jointly revealed positive associations of bacteria with the digestion of various substrates (*Prevotella*) (17, 35), the degradation of fiber (Treponema) (36, 37), the biosynthesis of amino acids and fatty acids (*Christensenellaceae R-7 group*) (38), the production of succinic acid (*Succinivibrio*, *Succinivibrionaceae*), the production of propionate (*Succiniclasticum*) (39), the production of butyrate (*Lachnospiraceae*, *Ruminococcaceae*, and *Pseudobutyrivibrio*) (40), the degradation of starch (*Ruminobacter*), the metabolism of lipids (*Rikenellaceae RC9 gut group* and *Anaerovibrio*) (25, 41). The washed solid groups had more fermentation of beneficial microbiota, such as the *Rikenellaceae RC9 gut group*, *Prevotella*, and *Succinivibrionaceae UCG-002*. Additionally, there was no significant change in the constant gas production rate with or without the rumen solid washing, suggesting that the interaction of certain bacterial groups in the washing group may reduce rumen gas production. These results suggest that the combination of autoclaved fluid and fresh solid phases may be far superior to those of the direct inoculation of rumen contents and that the combination of different components has profound significance in biological applications. At the same time, it reflects the important research value of different matrices for the enrichment cultures of bacteria in the rumen solid phase.

The first limitation of this study is its small sample size (4 to 5 per group). However, it still showed good results on the effects of the different fractions of rumen contents on the fermentation of diets, providing insights for future *in vivo* studies. Second, biological organisms are complex and still need to be validated by *in vivo* inoculation experiments. In the future, metagenomic and/or metabolomic analyses for rumen microbiota should be performed. Despite these limitations, we confirmed that recombinations of the rumen fluid, solid phase, and solid washing solution had impacts on *in vitro* fermentation. The four groups of AFFS, FFFS, FFFW, and FFAS had good results of *in vitro* fermentation, and the effect of AFFS was the most significant.

In conclusion, *in vitro* experimentation revealed the significant differences in the efficiency of the fermentation of diets via recombined bacterial communities from the rumen contents. Bacteria from the rumen solid phase had the best *in vitro* fermentation results. These were followed by those of the rumen fluid and those of the washed solid. After the substrate was fermented with bacteria from the rumen solid, the cellulolytic bacteria, especially the succinic acid-producing bacteria, were boosted to improve the fermentation efficiency. Autoclaving effects on the fraction could affect fermentation and could increase harmful bacteria. However, the combination of bacteria from the solid phase and autoclaved fluid phase could enhance the fermentation efficiency of the bacteria from the solid phase, resulting in the AFFS group having the most ideal fermentation product amount, fermentation speed, and microflora structure. Therefore, the results of this study deepen our understanding of the post-fermentation characteristics and functions of bacteria from different parts of the rumen and provide a certain reference for the transplantation of rumen microbiota.

## MATERIALS AND METHODS

This study was performed following the protocols approved by the Animal Ethics Committee of the Institute of Feed Research of Chinese Academy of Agricultural Sciences (Protocol number: IFR-CAAS20220615). The experimental work was conducted at the Nankou pilot base of the Chinese Academy of Agricultural Sciences, Beijing, China (latitude, 40°13' N; longitude 116°06' E).

### Animals, feeding, and management.

Five adult male *Hu* sheep, weighing 41.70 ± 0.91 kg, aged about 1.5 years, and equipped with permanent rumen cannulas (internal diameter of 10 cm), were selected as rumen content donors. Each sheep was housed in a pen of 0.85 m in width and 3.4 m in length with a feeder, bucket, and slatted floor. All of the pens were disinfected and cleaned once a week. The sheep were fed twice a day at 8:00 and 16:00 and were given free access to water. The basal diets were formulated according to NRC (42), and its ingredients and chemical composition are shown in Table S1. The rumen contents were collected on the morning of the sixteenth day, after the sheep were fed the basal diets for 15 days.

### *In vitro* experiments.

A 2 × 4 factorial arrangement was selected. One factor is the rumen fluid phase, which included two levels: fresh fluid (FF) and autoclaved FF (AF). The other factor is the rumen solid phase, which included four levels: fresh solid (FS), autoclaved FS (AS), fresh solid washing solution (FW), and autoclaved FW (AW). The combinations of rumen fluid and solid phases were used for *in vitro* fermentation experiments. The details of the experimental design and the treatment process of the rumen contents are shown in Fig. 1A. The specific collection and preparation processes were as follows. Rumen contents were collected from 5 donor sheep in equal proportions 2 h after morning feeding, were mixed immediately, and were transported to the laboratory in a prewarmed, sealed, CO_2_-filled flask. The FF and FS were obtained via filtering through 4 layers of medical gauze under constant CO_2_ flushing. Next, to obtain FW containing the microbiota of the solid fraction, one aliquot of FS was mixed with a saline solution (0.9%) in a mass ratio of 25:75. The mixture was homogenized by a homogenizer for 5 min and was then squeezed through 4 layers of medical gauze. Then, a portion of the FF, FS, and FW were autoclaved at 115°C for 30 min to destroy all viable microbes while maintaining the fermentation products in order to get autoclaved FF (AF), autoclaved FS (AS), and autoclaved FW (AW). Finally, the 2 levels of the rumen fluid phase (FF and AF) were recombined with the 4 levels of the rumen solid phase (FS, AS, FW, and AW). In brief, FF and FS, FF and AS, AF and FS, as well as AF and AS were mixed in a mass ratio of 75:25. The group aliases were called FFFS, FFAS, AFFS, and AFAS, respectively. As the FW and AW were diluted 3 times, FF and FW, FF and AW, AF and FW, as well as AF and AW were mixed in a mass ratio of 50:50. The group aliases were called FFFW, FFAW, AFFW, and AFAW, respectively.

The artificial saliva was prepared anaerobically, as described by Menke (43). Each fermentor was filled with 30 mL of ruminal inoculum, which was prepared as a 1:2 ratio of the aforementioned rumen content and artificial saliva as a culture fluid. For each treatment, six replicates with diets (fermentation substrate, dried at 60°C for 48 h and milled through a 1 mm sieve) and three blanks without feed were prepared to correct for background gas production. A total of 200 mg diet that was the same for the feeding donors (ingredients and chemical composition are shown in Table S1) were transferred with a small spoon into a glass syringe (D-89173, Häberle Labortechnik, Germany) and were preheated at 39°C in an electric thermostatic water tank (CU-600, Bluepard, China). After the preparation of the aforementioned fermentors, the inlet end of the glass culture tube was discharged vertically upwards, despite the gas inside, the front silicone rubber tube was clamped with an iron clamp, and the corresponding initial scale value (mL) was recorded. The culture tube was quickly transferred into the preheated (39°C) thermostatic water bath oscillator (DSHZ-300A, Beijing ChengMeng Technology Company, China), and then the gas production was measured at 3, 6, 9, 12, 18, and 24 h. After 24 h, the fermentors were taken out and put into an ice water bath to stop the fermentation. The pH of the fermentation broth was measured immediately using a pH meter (testo2, Germany). The fermentation broth in the culture tube was drained into a 5 mL plastic centrifuge tube. Then, a portion of the fermentation broth was squeezed through 4 layers of gauze, transferred into the sterilized centrifuge tubes, and stored at −20°C for the analysis of the VFAs. The VFAs were determined via gas chromatography (GC), as described by Cao and Yang (44). The ammonia-N concentration was assessed via the phenol-sodium hypochlorite colorimetric method, as described by Wang (45). The microbial crude protein (MCP) was measured via the trichloroacetic acid precipitation method, according to Nandakumar (46). Another portion of the fermentation broth was collected and stored in microcentrifuge tubes in a −80°C freezer for the bacterial community determinations with 16S rRNA gene sequencing. However, due to the amount of fermentation broth, 11 samples were removed for sequencing.

### DNA extraction and high-throughput sequencing.

Microbial DNA was extracted from the fermentor samples using a PowerSoil DNA Isolation Kit (MoBio Laboratories, Carlsbad, CA). The DNA integrity was evaluated using agarose gel electrophoresis. The concentration of the genomic DNA was assessed using a NanoDrop spectrophotometer (Thermo Scientific). The V3-4 hypervariable region of the bacterial 16S rRNA gene was amplified with the primers 338F (5′-ACTCCTACGGGAGGCAGCAG-3′) and 806R (5′-GGACTACNNGGGTATCTAAT-3′). The PCR was carried out on a MasterCycler Gradient (Eppendorf, Germany) using 25 μL reaction volumes that contained 12.5 μL 2× *Taq* PCR MasterMix, 3 μL BSA (2 ng/μL), 1 μL forward primer (5 μM), 1 μL reverse primer (5 μM), 2 μL template DNA, and 5.5 μL ddH_2_O. The cycling parameters were 95°C for 5 min, 28 cycles of 95°C for 45 s, 55°C for 50 s, and 72°C for 45 s, with a final extension at 72°C for 10 min. The PCR products were purified using an Agencourt AMPure XP Kit (Beckman Coulter, La Brea, CA, United States). Purified amplicons were used to generate the library and were sequenced on an Illumina MiSeq-PE300 platform at Beijing Allwegene Technology Co., Ltd. (Beijing, China).

Raw sequences obtained from the Illumina sequencer were merged using Flash (v1.20) (47). Afterward, Flash and Pear were used to merge the sequences at both ends. According to the PE overlap correlation, the minimum overlap was set to 10 bp, and the mismatch rate was set to 0.1, allowing for fasta sequences to be obtained. To generate high-quality reads, Trimmomatic (v0.36) (SLIDINGWINDOW:50:20 and MINLEN:120) was used to trim adapters and filter sequences, with the phred score parameter set over 20 and the length parameter set over 120 bp. In addition, the sequences containing N were removed by Pear (v0.9.6) (48). Finally, chimeric sequences were filtered using the USEARCH software package, based on the UCHIME algorithm (49). The unknown database was removed by *de novo* means. Simultaneously, the short sequences that failed to meet the requirements were removed, and high-quality sequences of clean reads were obtained. Qualified reads were clustered into operational taxonomic units (OTUs) at a similarity level of 97% using the UPARSE algorithm of the Quantitative Insight into Microbial Ecology (QIIME v 1.9.1) tool kit. The Ribosomal Database Project (RDP) Classifier tool was used to classify all of the sequences into different taxonomic groups against the SILVA138 database. Additionally, the alpha diversities (observed species and Shannon index) and the beta diversities, based on Bray-Curtis dissimilarity matrices, were calculated. An analysis of similarities (ANOSIM) was used to examine the statistically significant differences in beta diversity.

### Statistical analysis.

The cumulative gas values were fitted with the following exponential model without a lag phase as described by France (50):

$$\text{Y }=\text{ Vmax }\times \;\left(\text{1 }-{\text{ exp}}^{-\text{KT}}\right)$$

,where Y is the cumulative gas volume (mL) produced at time T (h), Vmax is the asymptotic gas volume (mL), K is the constant gas production rate of the fermentation substrate (mL/h), and T is the incubation time (h).

The data of the ruminal fermentation parameters were analyzed using IBM SPSS Statistics 26 (IBM Corp., Armonk, NY, USA). A GLM model used was as follows:

$${\text{Y}}_{\text{ijk}}{=\mu +\text{F}}_{\text{i}}{+\text{S}}_{\text{j}}{+\text{FS}}_{\text{ij}}{+\varepsilon }_{\text{ijk}}$$

,where Y_ijk_ is the dependent variable, μ is the overall mean, F_i_ is the fixed effect of the fluid portion of the rumen contents, S_j_ is the fixed effect of the solid portion of the rumen contents, FS_ij_ is the interaction effect of the fluid and solid portions, and ε_ijk_ is the random residual error. Statistically significant differences were detected, and the means were compared via the Holm-Sidak multiple comparisons correction test. A *P* value of <0.05 was considered to be indicative of a statistically significant result.

The alpha diversity of the rumen microbial data between treatments was tested via the Kruskal-Wallis test, and a *post hoc* Dunn’s test for multiple comparisons with a Bonferroni adjustment was used to evaluate the differences between treatments. Boxplots were made in R (“ggpubr” package). Beta diversity was visualized via a PCoA plot. The featured bacteria among the treatments were identified via the linear discriminant analysis (LDA) effect size (LEfSe) with an LDA score of >3 (51). A network analysis of the bacteria, using the R package “psych” and the Spearman correlations (*r*), was used to reveal the correlations between the rumen bacteria at the genus level. The Gephi software package was used for visualization. The Spearman correlations were also calculated to assess the relationships between the microbial taxa abundance and phenotype data. The bacterial metabolic potentials and functions were predicted using the PICRUSt2 software package combined with the KEGG (Kyoto Encyclopedia of Genes and Genomes) database (52).

### Data availability.

The data sets in this study are available in the NCBI BioProject database, under the BioProject ID PRJNA861807.
