# Supplementary material for: In Vitro Modulation of Rumen Fermentation by Microbiota from the Recombination of Rumen Fluid and Solid Phases
Source: Microbiol Spectr. 2022 Dec 8;11(1):e03387-22. doi: 10.1128/spectrum.03387-22 (PMC9927485; doi:10.1128/spectrum.03387-22)
Supplement: Supplemental file 1 — Supplemental material. Download spectrum.03387-22-s0001.pdf, PDF file, 2.4 MB [file spectrum.03387-22-s0001.pdf]

Table S1. The ingredients and chemical composition of basal diets (%, as DM basis).

Ingredients	Formula	Nutrients[2]	Contents
corn	34.0	DM	77.88
Soybean meal	10.0	ME, MJ/kg	10.01
Bran	12.0	CP	12.89
Premix [1]	4.00	EE	2.14
Corn stalk	20.0	NDF	35.96
Whole corn silage	20.0	ADF	18.46
Total	100	Ash	9.71
		Ca	1.11
		P	0.31

Abbreviations: DM, dry matter; ME, Metabolizable energy; CP, crude protein; EE, ether extract; NDF, neutral detergent fiber; ADF, acid detergent fiber.

[1] The premix provides per kg of diet: vitamin A, 7000 IU; vitamin D3, 2480 IU; vitamin E, 500 mg; nicotinic acid, 15 mg; biotin, 0.05 mg; Cu, 8 mg; Fe, 40 mg; Mn, 20 mg; Zn, 40 mg; I, 0.4 mg; Se, 0.4 mg; Co, 0.3 mg; NaCl, 2.4 g; Ca, 3.2 g; P, 0.48 g.

[2] Nutrients were all measured values except metabolizable energy, which was calculated by the equations from NRC (1).

Table S2. Effects of different combination of rumen fluid and solid phases on gas production *in vitro*

Items[1]	FF				AF				SEM	*P*-value		
	FS	AS	FW	AW	FS	AS	FW	AW		Fluid	Solid	F×S
3 hours	37.80^{b}	31.13^{c}	16.13^{c}	19.40^{c}	52.10^{a}	1.00^{d}	20.80^{c}	1.00^{d}	0.667	<0.001	<0.001	<0.001
6 hours	58.40^{b}	50.75^{c}	29.88^{de}	32.80^{de}	74.70^{a}	28.33^{e}	35.80^{d}	6.75^{f}	0.803	<0.001	<0.001	<0.001
9 hours	70.40^{b}	59.88^{c}	34.38^{d}	38.00^{d}	86.70^{a}	37.00^{d}	40.40^{d}	14.00^{e}	0.922	<0.001	<0.001	<0.001
12 hours	80.80^{b}	68.88^{c}	42.88^{d}	41.60^{d}	94.90^{a}	43.00^{d}	43.80^{d}	17.75^{e}	1.086	<0.001	<0.001	<0.001
18 hours	92.60^{b}	80.88^{c}	46.63^{d}	46.80^{d}	103.90^{a}	47.00^{d}	48.40^{d}	20.00^{e}	1.136	<0.001	<0.001	<0.001
24 hours	98.40^{ab}	87.88^{b}	51.13^{c}	50.20^{c}	108.90^{a}	48.33^{c}	50.40^{c}	20.75^{d}	1.304	<0.001	<0.001	<0.001

[1]V_{12h} gas production at 12 hours; Vmax, the asymptotic gas volume; K, the constant gas production rate; MCP, Microbial crude protein; TVFA, total volatile fatty acids; APratio, acetate to propionate ratio. Each group includes two factors, one factor is rumen fluid phase, which included two levels: fresh fluid (FF) and autoclaved FF (AF); The other factor is rumen solid phase, which included four levels: fresh solid (FS), autoclaved FS (AS), fresh solid washing solution (FW), and autoclaved FW (AW).

SEM=standard error of the means.

[a, b, c, d, e, f] Means in the same row with different superscripts indicate significant difference ($P < 0.05$).

Table S3. Effects of different combination of rumen fluid and solid phases on fermentation parameters *in vitro*

Items[1]	FF				AF				SEM	*P*-value		
	FS	AS	FW	AW	FS	AS	FW	AW		Fluid	Solid	F×S
V_{12h}, mL	75.50^{b}	65.22^{c}	38.34^{d}	39.86^{d}	89.65^{a}	38.08^{d}	41.85^{d}	14.06^{e}	0.458	0.060	<0.001	<0.001
Vmax, mL	100.50^{ab}	90.93^{b}	53.62^{c}	50.54^{c}	107.17^{a}	56.59^{c}	50.22^{c}	29.83^{d}	1.449	<0.001	<0.001	<0.001
K, mL/h	0.14^{bc}	0.13^{bc}	0.13^{c}	0.17^{ab}	0.20^{a}	0.10^{cd}	0.19^{a}	0.07^{d}	0.005	0.789	0.001	<0.001
pH	5.57^{d}	5.56^{d}	6.51^{a}	6.50^{a}	5.44^{e}	5.70^{c}	6.32^{b}	6.33^{b}	0.007	<0.001	<0.001	<0.001
NH_3-N, mg/dl	42.40^{ab}	41.93^{ab}	35.68^{c}	36.91^{bc}	45.88^{a}	27.99^{d}	32.63^{cd}	21.43^{e}	0.648	<0.001	<0.001	<0.001
MCP, mg/ml	3.60^{bc}	5.55^{a}	4.50^{ab}	4.82^{ab}	4.22^{ab}	2.47^{cd}	3.63^{bc}	1.52^{d}	0.163	<0.001	0.195	<0.001
Lactate, mmol/L	0.05^{c}	0.20^{c}	0.00^{c}	0.25^{c}	0.05^{c}	46.25^{a}	0.05^{c}	18.38^{b}	0.560	<0.001	<0.001	<0.001
Formate, mmol/L	0.62^{c}	0.61^{c}	0.64^{c}	0.66^{c}	0.72^{c}	7.85^{a}	0.61^{c}	2.51^{b}	0.151	<0.001	<0.001	<0.001
TVFA, mmol/L	120.69^{ab}	115.40^{b}	44.43^{cd}	53.66^{c}	132.99^{a}	43.44^{cd}	55.28^{c}	28.71^{d}	1.928	<0.001	<0.001	<0.001
Acetate, mmol/L	68.11^{a}	64.54^{a}	25.70^{ab}	30.80^{b}	66.70^{a}	24.38^{bc}	29.94^{b}	17.73^{c}	1.150	<0.001	<0.001	<0.001
Propionate, mmol/L	33.01^{b}	32.27^{b}	9.64^{de}	11.86^{d}	40.79^{a}	11.09^{d}	16.33^{c}	6.06^{e}	0.449	0.002	<0.001	<0.001
Butyrate, mmol/L	13.85^{b}	13.11^{b}	6.33^{c}	7.67^{c}	17.79^{a}	6.07^{c}	5.82^{c}	3.48^{d}	0.272	0.001	<0.001	<0.001
Valerate, mmol/L	2.71^{b}	2.47^{b}	1.03^{cd}	1.29^{cd}	3.67^{a}	0.85^{d}	1.50^{c}	0.81^{d}	0.060	0.176	<0.001	<0.001
Isobutyrate, mmol/L	1.27^{b}	1.23^{b}	0.70^{cd}	0.72^{cd}	1.54^{a}	0.65^{cd}	0.86^{c}	0.61^{d}	0.024	<0.001	<0.001	<0.001
Isovalerate, mmol/L	1.74^{b}	1.78^{b}	1.03^{cd}	1.33^{c}	2.50^{a}	0.39^{e}	0.82^{d}	0.38^{e}	0.042	<0.001	<0.001	<0.001
APratio	2.06^{cd}	1.99^{de}	2.66^{b}	2.59^{b}	1.63^{f}	2.20^{c}	1.83^{e}	2.89^{a}	0.023	<0.001	<0.001	<0.001

[1]V_{12h} gas production at 12 hours; Vmax, the asymptotic gas volume; K, the constant gas production rate; MCP, Microbial crude protein; TVFA, total volatile fatty acids; APratio, acetate to propionate ratio. Each group includes two factors, one factor is rumen fluid phase, which included two levels: fresh fluid (FF) and autoclaved FF (AF); The other factor is rumen solid phase, which included four levels: fresh solid (FS), autoclaved FS (AS), fresh solid washing solution (FW), and autoclaved FW (AW).

SEM=standard error of the means.

$^{a, b, c, d, e, f}$ Means in the same row with different superscripts indicate significant difference ($P < 0.05$).

Table S4. Effects of different combination of rumen fluid and solid phases on gas production *in vitro*

Items[1]	FF				AF				SEM	*P*-value		
	FS	AS	FW	AW	FS	AS	FW	AW		Fluid	Solid	F×S
Observed species	949.60^{ab}	1044.98^{a}	1056.00^{a}	1018.32^{a}	860.60^{b}	102.00^{c}	1037.56^{a}	96.00^{c}	11.780	<0.001	<0.001	<0.001
Shannon Index	6.18^{ab}	6.61^{ab}	6.79^{a}	6.41^{ab}	6.09^{b}	2.21^{c}	6.72^{a}	2.33^{c}	0.070	<0.001	<0.001	<0.001

[1]V_{12h} gas production at 12 hours; Vmax, the asymptotic gas volume; K, the constant gas production rate; MCP, Microbial crude protein; TVFA, total volatile fatty acids; APratio, acetate to propionate ratio. Each group includes two factors, one factor is rumen fluid phase, which included two levels: fresh fluid (FF) and autoclaved FF (AF); The other factor is rumen solid phase, which included four levels: fresh solid (FS), autoclaved FS (AS), fresh solid washing solution (FW), and autoclaved FW (AW).

SEM=standard error of the means.

[a, b, c, d, e, f] Means in the same row with different superscripts indicate significant difference ($P < 0.05$).

Table S5. Effects of different combination of rumen fluid and solid phases on composition of microbiota at the genus level.

Items[1]	FF				AF				SEM	*P*-value		
	FS	AS	FW	AW	FS	AS	FW	AW		Fulid	Solid	F×S
Prevotella	36.26^{ab}	38.59^{a}	32.09^{ab}	41.08^{a}	28.18^{b}	2.18^{c}	27.45^{b}	0.48^{c}	15.130	<0.001	<0.001	<0.001
Succinivibrio	19.11^{b}	10.80^{c}	11.68^{c}	10.93^{c}	31.01^{a}	0.71^{d}	15.95^{bc}	0.56^{d}	9.981	0.536	<0.001	<0.001
Streptococcus	0.03^{c}	0.01^{c}	0.02^{c}	0.01^{c}	0.21^{c}	45.20^{b}	0.16^{c}	62.46^{a}	23.002	<0.001	<0.001	<0.001
Rikenellaceae_RC9_gut_group	5.78^{c}	9.14^{a}	9.58^{a}	8.13^{ab}	3.91^{d}	0.00^{e}	6.62^{bc}	0.00^{e}	3.520	<0.001	0.001	<0.001
Prevotellaceae_YAB2003_group	0.07^{c}	0.05^{c}	0.05^{c}	0.07^{c}	0.10^{c}	37.00^{a}	0.32^{c}	6.74^{b}	11.034	0.001	0.001	0.001
Succiniclasticum	4.84^{a}	3.71^{abc}	4.28^{ab}	2.96^{bcd}	2.56^{cd}	0.00^{e}	2.10^{d}	0.01^{e}	1.839	<0.001	<0.001	0.121
Christensenellaceae_R-7_group	3.58^{a}	3.45^{ab}	2.52^{bc}	2.72^{abc}	2.12^{c}	0.00^{d}	2.33^{c}	0.00^{d}	1.356	<0.001	<0.001	<0.001
Veillonellaceae_UCG-001	1.81^{bc}	1.34^{cd}	2.86^{a}	2.40^{ab}	0.94^{d}	0.00^{e}	1.75^{bc}	0.00^{e}	1.030	<0.001	<0.001	0.032
Clostridium_sensu_stricto_1	0.00^{c}	0.00^{c}	0.00^{c}	0.00^{c}	0.02^{c}	4.07^{b}	0.03^{c}	8.45^{a}	3.424	<0.001	<0.001	<0.001
Prevotellaceae_UCG-003	1.14^{bc}	0.81^{bcd}	2.38^{a}	3.04^{a}	0.60^{cd}	0.00^{d}	1.51^{b}	0.00^{d}	1.131	<0.001	0.264	0.027
Treponema	2.71^{a}	2.49^{a}	0.35^{b}	0.28^{b}	2.80^{a}	0.00^{b}	0.98^{b}	0.00^{b}	1.327	0.212	0.008	0.074
Prevotellaceae_UCG-001	1.20^{b}	1.82^{a}	1.71^{a}	1.90^{a}	0.89^{b}	0.00^{c}	1.02^{b}	0.01^{c}	0.698	<0.001	0.005	<0.001
Ruminobacter	0.04^{b}	0.02^{b}	0.23^{b}	0.48^{b}	0.62^{b}	0.00^{b}	6.75^{a}	0.00^{b}	3.060	0.086	0.066	0.047
Selenomonas	0.62^{c}	0.42^{cd}	0.59^{c}	0.67^{c}	1.86^{b}	2.45^{a}	1.77^{b}	0.05^{d}	0.800	<0.001	0.076	0.115
Fretibacterium	0.22^{d}	0.26^{d}	3.01^{a}	2.46^{b}	0.03^{d}	0.00^{d}	0.84^{c}	0.00^{d}	1.178	0.001	0.567	0.526
Ruminococcus	1.37^{ab}	1.05^{b}	0.33^{c}	0.34^{c}	1.45^{a}	0.00^{c}	1.16^{ab}	0.00^{c}	0.607	0.372	<0.001	<0.001
Succinivibrionaceae_UCG-002	0.18^{b}	0.10^{b}	0.80^{b}	0.70^{b}	1.68^{a}	0.00^{b}	2.05^{a}	0.00^{b}	0.923	0.022	<0.001	<0.001
Veillonella	0.00^{b}	0.00^{b}	0.00^{b}	0.00^{b}	0.00^{b}	0.06^{b}	0.00^{b}	6.27^{a}	2.660	0.030	0.030	0.030
Anaerovibrio	0.35^{c}	0.30^{cd}	1.09^{b}	1.43^{a}	0.46^{c}	0.00^{d}	1.33^{ab}	0.03^{d}	0.574	0.041	0.063	0.002
Lachnospiraceae NK3A20 group	0.80^{b}	0.96^{ab}	0.60^{b}	0.65^{b}	0.77^{b}	0.02^{c}	1.32^{a}	0.00^{c}	0.492	0.064	<0.001	<0.001
Lachnoclostridium	0.31^{c}	0.48^{c}	1.18^{ab}	0.54^{c}	0.24^{c}	0.39^{c}	0.73^{bc}	1.35^{a}	0.504	0.544	0.430	0.069
NK4A214_group	0.74^{a}	0.89^{a}	0.85^{a}	0.82^{a}	0.41^{b}	0.00^{c}	0.82^{a}	0.00^{c}	0.392	<0.001	0.002	<0.001

[1]Each group includes two factors, one factor is rumen fluid phase, which included two levels: fresh fluid (FF) and autoclaved FF (AF); The other factor is rumen solid phase, which included four levels: fresh solid (FS), autoclaved FS (AS), fresh solid washing solution (FW), and autoclaved FW (AW).

Table S6. Analysis of similarities (ANOSIM) output for beta diversity

Comparison1	R	*P*-value
FFFS vs FFAS	0.48	0.024
FFFS vs AFFS	0.90	0.006
FFFS vs AFAS	1.00	0.010
FFFS vs FFFW	0.84	0.011
FFFS vs FFAW	1.00	0.009
FFFS vs AFFW	1.00	0.010
FFFS vs AFAW	1.00	0.012
FFAS vs AFFS	0.94	0.008
FFAS vs AFAS	1.00	0.025
FFAS vs FFFW	0.71	0.007
FFAS vs FFAW	0.97	0.007
FFAS vs AFFW	0.93	0.012
FFAS vs AFAW	1.00	0.030
AFFS vs AFAS	1.00	0.005
AFFS vs FFFW	1.00	0.004
AFFS vs FFAW	1.00	0.008
AFFS vs AFFW	1.00	0.010
AFFS vs AFAW	1.00	0.010
AFAS vs FFFW	1.00	0.007
AFAS vs FFAW	1.00	0.009
AFAS vs AFFW	1.00	0.007
AFAS vs AFAW	0.82	0.027
FFFW vs FFAW	0.44	0.036
FFFW vs AFFW	0.79	0.012
FFFW vs AFAW	1.00	0.006
FFAW vs AFFW	0.94	0.011
FFAW vs AFAW	1.00	0.006
AFFW vs AFAW	1.00	0.008

[1] FFFS = fresh fluid (FF) + fresh solid (FS); FFAS = fresh fluid (FF) + autoclaved solid (AS); FFFW = fresh fluid (FF) + fresh solid washing solution (FW); FFAW = fresh fluid (FF) + autoclaved solid washing solution (AW); AFFS = autoclaved fluid (AF) + fresh solid (FS); AFAS = autoclaved fluid (AF) + autoclaved solid (AS); AFFW = autoclaved fluid (AF) + fresh solid washing solution (FW); AFAW = autoclaved fluid (AF) + autoclaved solid washing solution (AW).

Table S7. Functional predictions for rumen microbiota in eight treatments; only the marked KEGG pathways in the essay were shown.

Items[1]	FF				AF				SEM	*P*-value
	FS	AS	FW	AW	FS	AS	FW	AW		
**Metabolism**	78.72^{b}	79.34^{a}	79.36^{a}	79.54^{a}	77.85^{c}	78.15^{c}	78.67^{b}	77.84^{c}	0.116	<0.001
**Amino acid metabolism**	12.99^{a}	12.89^{ab}	12.89^{ab}	12.89^{ab}	12.71^{b}	12.05^{c}	12.72^{b}	12.70^{b}	0.049	0.003
Valine, leucine and isoleucine biosynthesis	2.27^{b}	2.21^{b}	2.26^{b}	2.23^{b}	2.28^{b}	2.01^{c}	2.28^{b}	2.41^{a}	0.020	0.004
Alanine, aspartate and glutamate metabolism	1.89^{a}	1.87^{a}	1.81^{b}	1.86^{ab}	1.85^{ab}	1.55^{c}	1.80^{b}	1.42^{d}	0.027	<0.001
Lysine biosynthesis	1.63^{b}	1.59^{b}	1.63^{b}	1.64^{b}	1.60^{b}	1.60^{b}	1.63^{b}	1.70^{a}	0.007	0.006
Phenylalanine, tyrosine and tryptophan biosynthesis	1.39^{bc}	1.37^{cd}	1.34^{d}	1.36^{cd}	1.39^{c}	1.43^{ab}	1.37^{cd}	1.46^{a}	0.007	<0.001
Cysteine and methionine metabolism	1.29^{b}	1.27^{bc}	1.25^{c}	1.25^{c}	1.29^{b}	1.44^{a}	1.26^{bc}	1.45^{a}	0.013	<0.001
Histidine metabolism	1.28^{bc}	1.28^{bc}	1.30^{b}	1.29^{bc}	1.25^{d}	1.29^{b}	1.25^{cd}	1.39^{a}	0.008	<0.001
Glycine, serine and threonine metabolism	1.18^{b}	1.18^{b}	1.18^{b}	1.18^{b}	1.16^{c}	1.23^{a}	1.16^{c}	1.18^{b}	0.004	<0.001
Arginine and proline metabolism	0.75^{a}	0.76^{a}	0.77^{a}	0.77^{a}	0.70^{c}	0.56^{e}	0.72^{b}	0.61^{d}	0.012	<0.001
Valine, leucine and isoleucine degradation	0.40^{c}	0.44^{b}	0.47^{a}	0.44^{b}	0.33^{d}	0.26^{e}	0.39^{c}	0.34^{d}	0.011	<0.001
Phenylalanine metabolism	0.38^{ab}	0.38^{ab}	0.37^{abc}	0.39^{a}	0.36^{bc}	0.20^{d}	0.35^{c}	0.18^{d}	0.013	<0.001
**Carbohydrate metabolism**	13.71^{cd}	13.85^{c}	13.57^{cd}	13.74^{cd}	13.40^{d}	14.47^{b}	13.75^{cd}	15.48^{a}	0.108	<0.001
C5-Branched dibasic acid metabolism	1.77^{bc}	1.64^{cd}	1.74^{c}	1.71^{c}	1.93^{a}	1.56^{d}	1.89^{ab}	1.95^{a}	0.026	0.001
Pentose phosphate pathway	1.50^{ab}	1.50^{abc}	1.48^{bc}	1.46^{cd}	1.53^{a}	1.43^{d}	1.53^{a}	1.50^{abc}	0.006	0.001
Amino sugar and nucleotide sugar metabolism	1.14^{bc}	1.17^{b}	1.10^{c}	1.13^{bc}	1.09^{c}	1.31^{a}	1.11^{c}	1.31^{a}	0.014	<0.001
Glycolysis / Gluconeogenesis	1.01^{c}	1.03^{c}	1.01^{c}	1.01^{c}	0.99^{c}	1.51^{b}	1.02^{c}	1.58^{a}	0.037	<0.001
Citrate cycle (TCA cycle)	1.14^{a}	1.14^{a}	1.15^{a}	1.15^{a}	1.10^{ab}	1.13^{ab}	1.09^{c}	0.95^{d}	0.011	<0.001
Galactose metabolism	1.00^{c}	1.06^{c}	0.99^{cd}	1.05^{c}	0.90^{d}	1.35^{b}	0.99^{cd}	1.47^{a}	0.031	<0.001
Starch and sucrose metabolism	1.07^{ab}	1.16^{a}	1.03^{bc}	1.09^{ab}	0.97^{c}	1.14^{a}	1.07^{ab}	1.14^{a}	0.013	0.002
Fructose and mannose metabolism	1.03^{cd}	1.07^{bc}	1.01^{cd}	1.06^{bc}	0.95^{e}	1.19^{a}	0.98^{de}	1.12^{b}	0.014	<0.001
Pyruvate metabolism	0.94^{cd}	0.94^{cd}	0.95^{cd}	0.92^{d}	0.94^{cd}	1.18^{b}	0.98^{c}	1.33^{a}	0.023	<0.001
Pentose and glucuronate interconversions	0.80^{ab}	0.84^{a}	0.78^{ab}	0.82^{ab}	0.71^{c}	0.28^{e}	0.76^{bc}	0.36^{d}	0.034	<0.001
**Metabolism of cofactors and vitamins**	14.59^{a}	14.33^{a}	14.30^{a}	14.63^{a}	14.52^{a}	12.92^{b}	14.18^{a}	12.13^{c}	0.143	0.001
One carbon pool by folate	2.03^{b}	2.04^{b}	1.97^{bc}	2.03^{b}	1.95^{c}	2.14^{a}	1.92^{c}	1.83^{d}	0.017	<0.001
Folate biosynthesis	1.51^{c}	1.44^{c}	1.44^{c}	1.47^{c}	1.63^{b}	1.85^{a}	1.45^{c}	1.66^{b}	0.024	<0.001

Items[1]	FF				AF				SEM	*P*-value
	FS	AS	FW	AW	FS	AS	FW	AW		
Thiamine metabolism	1.62^{a}	1.61^{a}	1.60^{a}	1.65^{a}	1.51^{b}	1.27^{c}	1.60^{a}	1.25^{c}	0.025	<0.001
Vitamin B6 metabolism	1.54^{ab}	1.46^{bc}	1.41^{c}	1.48^{bc}	1.59^{a}	1.43^{c}	1.44^{c}	1.24^{d}	0.018	<0.001
Nicotinate and nicotinamide metabolism	1.24^{abc}	1.22^{bc}	1.18^{c}	1.22^{bc}	1.29^{ab}	1.31^{a}	1.25^{abc}	1.07^{d}	0.013	0.001
Biotin metabolism	1.34^{bcd}	1.22^{d}	1.35^{bc}	1.24^{cd}	1.53^{a}	0.63^{f}	1.41^{ab}	0.85^{e}	0.048	<0.001
Lipoic acid metabolism	1.11^{b}	1.22^{ab}	1.16^{ab}	1.31^{a}	0.84^{c}	1.24^{ab}	0.94^{c}	1.12^{b}	0.031	0.001
Riboflavin metabolism	1.01^{ab}	0.97^{b}	0.99^{ab}	1.00^{ab}	1.04^{a}	0.55^{c}	1.01^{ab}	0.52^{c}	0.033	<0.001
Porphyrin and chlorophyll metabolism	0.64^{ab}	0.64^{ab}	0.66^{ab}	0.66^{a}	0.59^{b}	0.21^{d}	0.65^{ab}	0.33^{c}	0.027	<0.001
Ubiquinone and other terpenoid-quinone biosynthesis	0.63^{a}	0.62^{a}	0.61^{a}	0.64^{a}	0.66^{a}	0.34^{b}	0.60^{a}	0.24^{c}	0.025	0.002

[1]Each group includes two factors, one factor is rumen fluid phase, which included two levels: fresh fluid (FF) and autoclaved FF (AF); The other factor is rumen solid phase, which included four levels: fresh solid (FS), autoclaved FS (AS), fresh solid washing solution (FW), and autoclaved FW (AW).

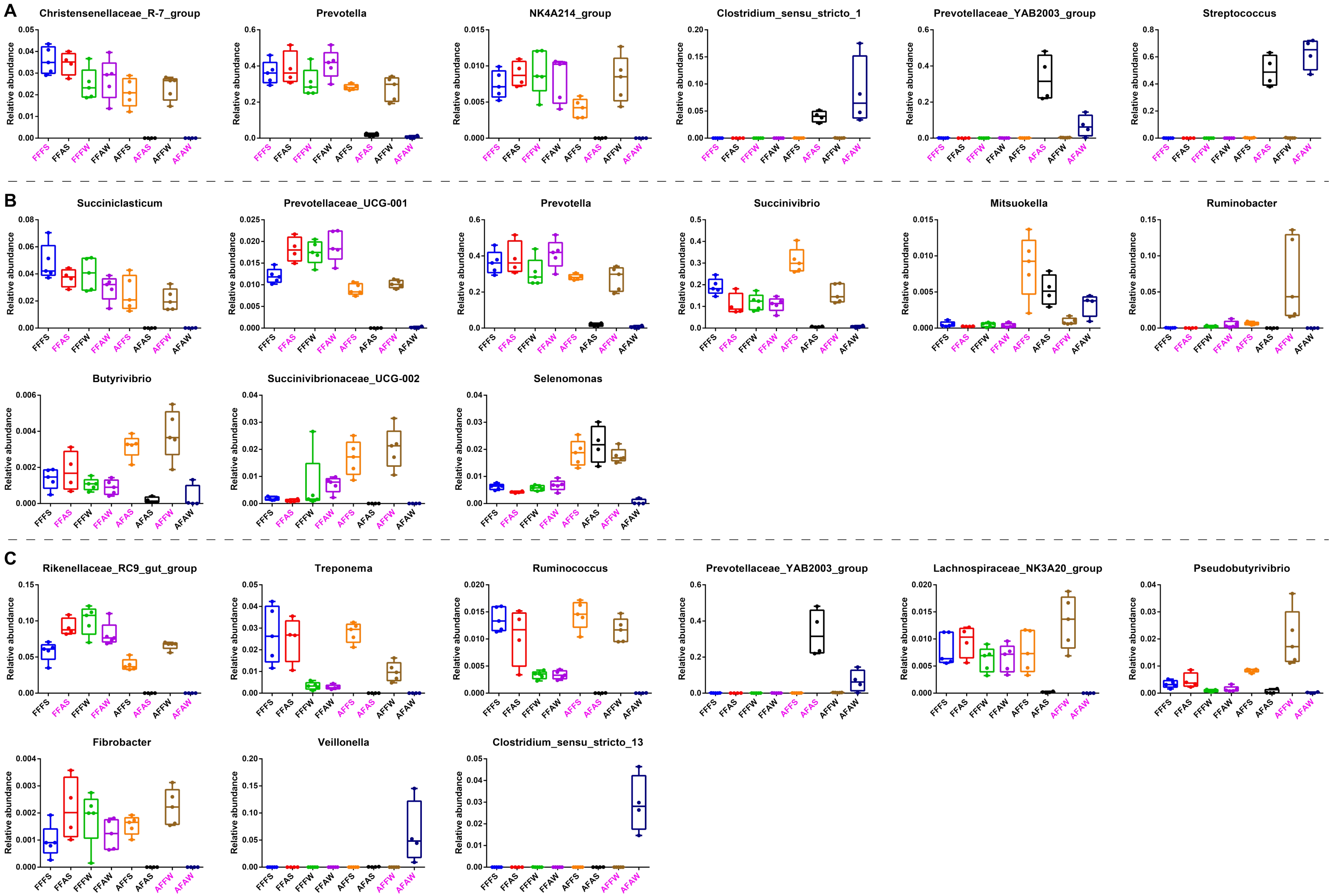
A
Christensenellaceae_R-7_group
Prevotella
NK4A214_group
Clostridium_sensu_stricto_1
Prevotellaceae_YAB2003_group
Streptococcus
B
Succiniclasticum
Prevotellaceae_UCG-001
Prevotella
Succinivibrio
Mitsuokella
Ruminobacter
Butyrivibrio
Succinivibrionaceae_UCG-002
Selenomonas
C
Rikenellaceae_RC9_gut_group
Treponema
Ruminococcus
Prevotellaceae_YAB2003_group
Lachnospiraceae_NK3A20_group
Pseudobutyrivibrio
Fibrobacter
Veillonella
Clostridium_sensu_stricto_13
Relative abundance
FFFS
FFAS
FFFW
FFAW
AFFS
AFAS
AFFW
AFAW

Figure S1. Main factors affecting the signature microbiota. **A.** Non-autoclaved vs all-autoclaved; **B.** fluid phase (F) vs solid phase (S) vs washing solution (W) **C.** fresh fluid (FF) vs autoclaved fluid (AF); fresh solid (FS) vs autoclaved solid (AS); fresh washing solution (FW) vs autoclaved washing solution (AW). FFFS = fresh fluid (FF) + fresh solid (FS); FFAS = fresh fluid (FF) + autoclaved solid (AS); FFFW = fresh fluid (FF) + fresh solid washing solution (FW); FFAW = fresh fluid (FF) + autoclaved solid washing solution (AW); AFFS = autoclaved fluid (AF) + fresh solid (FS); AFAS = autoclaved fluid (AF) + autoclaved solid (AS); AFFW = autoclaved fluid (AF) + fresh solid washing solution (FW); AFAW = autoclaved fluid (AF) + autoclaved solid washing solution (AW).

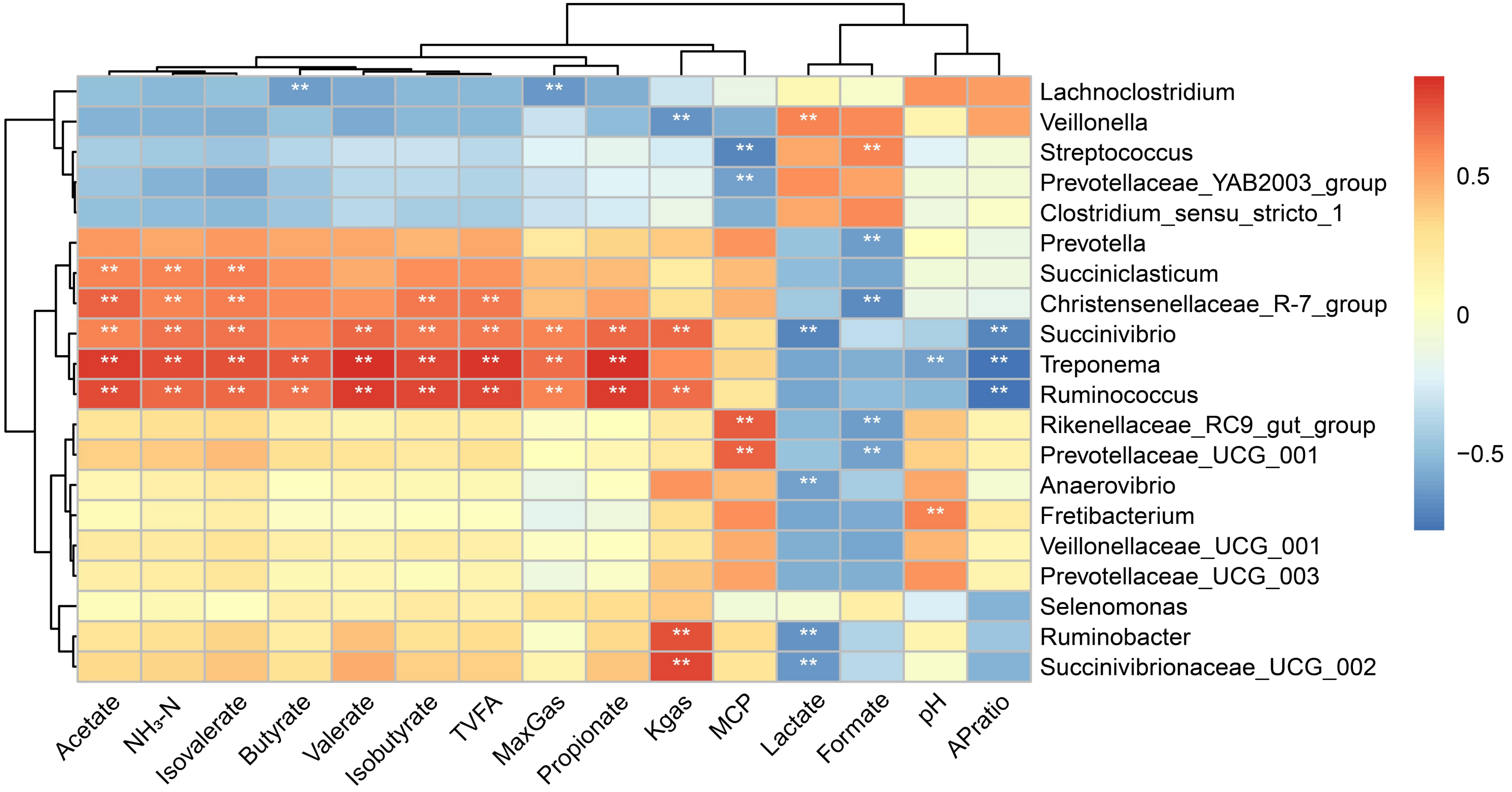

Figure S2. Correlation between the top 20 genera and fermentation parameters. Red color represents a positive correlation, while blue represents a negative correlation. Only strong (Spearman's |r| > 0.60) and significant (P < 0.05) correlations are presented in the figure. Strong correlations are indicated by red and blue colors with 1 indicating a perfect positive correlation (dark red) and −1 indicating a negative correlation (dark blue), whereas weak correlations are indicated by yellow colors. Spearman test, * $p < 0.05$, ** $p < 0.01$. TVFA, total VFA; MaxGas, asymptotic gas volume; Kgas, constant gas production rate; APratio, acetate to propionate ratio.

REFERENCE

1. NRC. 2007. Nutrient requirements of small ruminants: sheep, goats, cervids, and new world camelids. National Academies Press, Washington, D.C.
